# Artificial intelligence-assisted design and optimization of stimuli-responsive nanocarriers for smart drug delivery

**DOI:** 10.1016/j.mtbio.2026.103153

**Published:** 2026-04-22

**Authors:** Sara Yazdani, Mehrdad Mozaffarian, Gholamreza Pazuki, Naghmeh Hadidi

**Affiliations:** a Department of Chemical Engineering, Amirkabir University of Technology, No. 424, Hafez Ave., P.O. Box 15875-4413, Tehran, Iran; b Department of Clinical Research and EM Microscope, Pasteur Institute of Iran (PII), Tehran, 1316943551, Iran

**Keywords:** Artificial intelligence (AI), Stimuli-responsive nanostructures, Data-driven drug delivery, Smart nanocarriers

## Abstract

Stimuli-responsive nanostructures are a revolutionary breakthrough in the controlled delivery of drugs, allowing for their precise spatiotemporal control. These intelligent materials are designed to respond to internal stimuli (such as pH, redox gradients, enzymatic activity) or external cues (such as temperature, light, magnetic fields), thus offering greater flexibility and functionality in biomedical applications. Recent achievements have been aimed at the development of multi-stimuli-responsive systems, which utilize a combination of several stimuli to achieve sophisticated control, greater stability, and greater therapeutic accuracy in complex biological media. At the same time, the incorporation of artificial intelligence (AI) into the design and optimization of these nanostructures has brought about real, data-driven breakthroughs. Thus, supervised machine learning algorithms have been employed to predict the drug-loading efficiency and gene-delivery ability of lipid and polymeric nanoparticles based solely on their compositional characteristics, thus facilitating the rational selection of optimal formulations without the need for extensive experimental screening. Moreover, AI-based modeling tools have been shown to possess the capability to predict complete drug release profiles in response to varying pH or redox environments, thus enabling the pre-optimization of release kinetics tailored to specific pathological microenvironments. With the integration of patient-specific biological information such as genomic signatures and biomarker profiles, AI-assisted approaches also allow for the personalization of carrier composition and sensitivity to stimuli. This review offers a thorough examination of the latest developments in stimuli-responsive nanostructures and their integration with AI. This complementary combination is revolutionizing the way carriers are designed, shifting from trial-and-error methods to predictive and personalized drug delivery systems, thus propelling the development of next-generation precision nanomedicine.

## Highlights

• This review presents an overview of AI-assisted design and optimization of internal and external stimuli-responsive nanocarriers for advanced drug and gene delivery.
•. Multi-stimuli-responsive systems are analyzed with emphasis on AI-guided predictive modeling for precision biomedical applications.
•. Applications in cancer therapy, gene delivery, and theranostics demonstrate how data-driven approaches overcome biological barriers.
•. Emerging AI-integrated multifunctional platforms enable intelligent, microenvironment-responsive payload release.
•. Future directions focus on AI-enabled clinical translation and personalized nanomedicine.

## Graphical abstract

**Figure:**
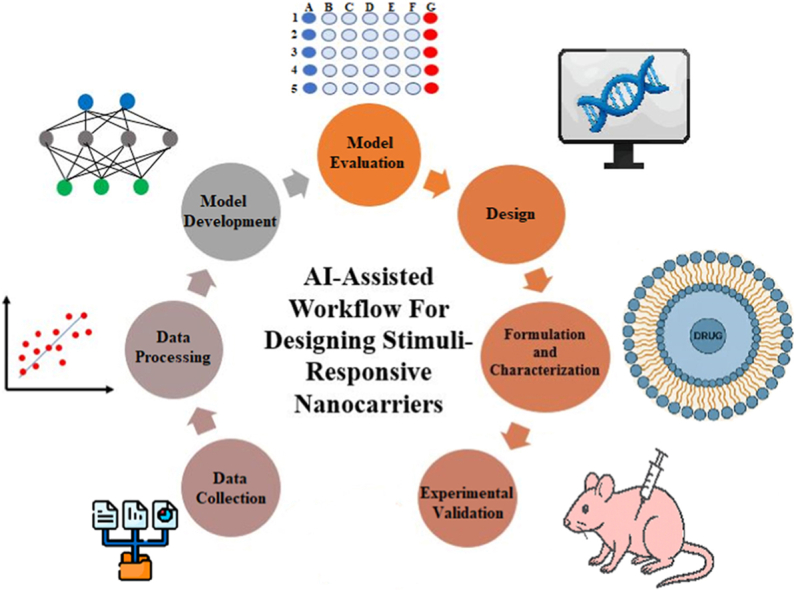

## 1 Introduction

Recent advancements in material science and drug delivery systems have led to the development of nanocarriers capable of achieving site- and time-specific cargo release. This progress has captured global interest, particularly in advancing stimuli-responsive delivery systems [1]. Stimuli-responsive nanocarriers are smart delivery systems engineered to undergo physicochemical or structural changes in response to specific internal (such as pH, redox potential, and enzymatic activity) or external (such as light, temperature, and magnetic fields) stimuli, thereby enabling controlled drug release [2,3]. This responsiveness can be achieved by integrating stimuli-responsive moieties into the nanocarrier structure. Such systems hold great promise for disease-specific treatment by offering controlled drug release, which can significantly reduce systemic side effects. Despite these promising prospects, several challenges still remain, including improving the precision of stimulus detection, enhancing drug loading efficiency, and ensuring patient safety [4]. Consequently, the development and optimization of stimuli-responsive nanocarriers continue to be a rapidly evolving area of research [2,4,5].

Stimuli-responsive nanostructures represent an advanced technological platform for targeted and controlled drug delivery in both pharmaceutical and biomedical fields [[6], [7], [8], [9]]. These nanocarriers possess distinctive and adjustable properties, enabling them to respond to a variety of endogenous and exogenous stimuli. Such responsiveness allows for the precise delivery of therapeutic agents to specific disease sites, improving drug efficacy, while minimizing adverse effects on healthy tissues [10,11]. Additionally, these smart systems can be designed to deliver multiple therapeutic agents simultaneously, such as chemotherapeutic drugs, immune checkpoint inhibitors, immune adjuvants, and enzymes. This multifunctional capability enhances therapeutic outcomes through synergistic effects and contributes to the establishment of long-term immune memory, which is crucial for preventing disease recurrence. By fine-tuning the sensitivity and release kinetics of these nanocarriers, researchers aim to develop more effective and personalized therapeutic strategies [11,12].

Stimuli‐responsive nanocarriers are increasingly being designed and optimized with the aid of AI tools. In fact, the convergence of AI with nanomedicine is revolutionizing carrier development, offering unprecedented precision and personalization [13,14]. Data‐driven approaches (machine learning (ML)/deep learning (DL)) allow high‐throughput *in silico* screening of materials and uncover complex structure–property relationships, effectively accelerating design cycles well beyond traditional trial‐and‐error methods [13,14]. For example, ML models can predict key performance metrics – such as drug encapsulation efficiency, release kinetics, and tissue distribution – directly from carrier formulation parameters [[15], [16], [17], [18]]. Such predictive models have even been shown to forecast entire drug‐release profiles and kinetic parameters under dynamic conditions, making formulation optimization much more efficient [13,19]. AI frameworks are also used to tune stimuli‐responsiveness: by designing nanomaterial structures (polymer or composite networks) that respond to specific triggers (pH, temperature, enzymes) with logic-gated release behaviors [17,20,21]. Integrated, closed‐loop pipelines – combining microfluidic synthesis, high‐throughput screening, and ML‐guided iteration – further refine carrier properties in feedback fashion, rapidly improving targeting accuracy, bioavailability, and therapeutic efficacy [14]. Crucially, these data‐driven methods enable personalized drug delivery: by incorporating patient-specific biological data (genomics, biomarkers, wearable sensor outputs), AI‐guided design can tailor carrier composition and release kinetics to individual physiological conditions [[22], [23], [24]]. Together, these advances illustrate how AI-enabled nanocarriers are faster to develop, more predictive in performance (including release kinetics and responsiveness), and ultimately capable of next-generation precision, patient-tailored therapy [[25], [26], [27], [28]].

This review provides a comprehensive examination of the latest developments in stimuli-responsive nanostructures, focusing on the underlying principles that govern their responsiveness and the mechanisms by which they achieve controlled drug release. It explores how these advanced systems interact with internal triggers such as pH shifts, redox gradients, and enzymatic activity, as well as external stimuli such as temperature, light, and magnetic fields. Moreover, the review highlights the diverse biomedical applications of these nanostructures, including their role in targeted drug delivery, gene therapy, and cancer treatment. Particular attention is given to the emerging role of AI and ML in accelerating the design, prediction, and optimization of these smart systems. By integrating data-driven approaches with material science, AI is enabling more precise control over release kinetics, responsiveness, and personalization of therapy. Ultimately, this review aims to present a forward-looking perspective on how AI-enhanced stimuli-responsive nanocarriers can revolutionize future strategies in precision medicine and advanced drug delivery.

## 2 Fundamentals of stimuli-responsive nanostructures

Stimuli-responsive nanostructures represent a pivotal innovation in the realm of advanced drug delivery and controlled release systems, distinguished by their ability to undergo physicochemical or structural transformations in response to specific triggers [29]. These nanostructures capitalize on their adaptability to achieve selective and controlled payload release, thereby enhancing therapeutic precision while minimizing off-target effects. Their design integrates a profound understanding of environmental interactions, enabling the development of systems that respond dynamically to diverse stimuli [29,30]. The stimuli that elicit these responses can be classified into two principal categories: internal stimuli and external stimuli. Internal stimuli are intrinsic to the biological microenvironment and include variations in pH levels, redox gradients, and the presence of specific enzymes. These triggers are often associated with pathological conditions such as tumor microenvironments, inflammatory sites, or intracellular compartments, making them ideal for selective drug release in diseased tissues [30]. Conversely, external stimuli are exogenous factors applied under controlled conditions, such as thermal inputs, electromagnetic fields, or optical signals. These external cues offer high spatial and temporal precision, allowing researchers to regulate the activation of nanostructures on demand [31,32]. Understanding the dual classification of these stimuli is fundamental to unraveling the potential of stimuli-responsive nanostructures in biomedical applications. To enable a clear and organized comparison between these two major categories, Table 1 summarizes the major features of internal and external stimuli-responsive nanostructures. Table 1 points out the source of activation, degree of spatial and temporal control, examples, and key advantages and limitations of these two major categories of stimuli-responsive nanostructures. This organized comparison enables a better understanding of the basic design principles and differences between autonomously activated systems and externally triggered platforms, thus helping in the rational development of smart nanocarriers for a specific biomedical application. The interplay between material properties and stimulus sensitivity forms the basis for the design of these advanced systems, paving the way for breakthroughs in targeted delivery, precision medicine, and theranostics approaches.

**Table 1:** Comparison of internal and external stimuli-responsive nanostructure.

External stimuli	Internal Stimuli	Feature
Applied physical triggers	Tumor microenvironment/biological signals	Source
On-demand activation	Autonomous activation	Control
High and externally adjustable	Limited and dependent on pathological localization	Spatial control
High and precisely controllable	Limited and biological regulated	Temporal control
Light, magnetic field, temperature	pH, redox, enzymes	Representative examples
Precise controllability, on-demand drug release, adjustable treatment parameters, suitable for theranostic	Disease-specific activation, Self-regulated release, reduced off-target toxicity, improved biological selectivity	Advantages
Need external equipment and specialized instrumentation	Biological heterogeneity and variability among patients	Limitation

### 2.1 Internal stimuli

Internal stimuli-responsive nanostructures have garnered significant attention in recent years due to their ability to leverage inherent physiological cues within the biological microenvironment for controlled therapeutic delivery. These systems are designed to exploit unique characteristics of pathological sites, such as tumors or inflamed tissues, where specific internal triggers such as pH gradients, redox potential, or enzymatic activity are markedly distinct from normal tissues [[33], [34], [35]]. By responding to these endogenous stimuli, nanostructures enable precise and localized release of therapeutic agents, minimizing side effects and enhancing therapeutic efficacy [33,36].

**pH-Responsive Systems:** The pH gradient between normal physiological environments (pH ∼7.4) and pathological sites, such as tumors (pH ∼6.5-6.8) or intracellular lysosomes and endosomes (pH ∼4.5-6.0), provides a robust internal trigger for drug delivery [37]. pH-responsive nanostructures are typically engineered using materials that exhibit pH-responsive behavior, such as ionizable polymers or acid-labile linkages. At lower pH levels, these systems undergo structural transformations, such as swelling, degradation, or charge reversal, leading to the release of encapsulated payloads. For example, polymeric micelles with acid-cleavable linkers can dissociate in acidic environments, ensuring the selective release of drugs at the tumor site or within lysosomal compartments [38,39]. Gao et al. [40] investigated the use of therapeutic ultrasound (US) for its ability to penetrate tissues deeply and provide controlled irradiation without causing radioactive damage. Although high-intensity focused ultrasound shows promise as a US-based therapy, its practical application is hindered by limited therapeutic outcomes and the potential to harm surrounding healthy tissues. In contrast, dynamic therapy, which uses low-intensity ultrasound, leverages sensitizers to produce reactive oxygen species for cancer treatment [40]. To overcome these challenges, the researchers developed pH-responsive nanoparticles (Ca@H) composed of hematoporphyrin monomethyl ether loaded onto CaCO_3_ [40]. Zhang et al. [41] addressed the challenges posed by tumor stromal microenvironments, which limit the efficacy of nanoparticle-based drug delivery by impeding nanoparticle transport. To overcome this, they [41] developed a pH-responsive drug delivery system using dihydralazine-loaded nanoparticles modified with cyclic RGD peptide-based prodrug of doxorubicin (cRGD-Dex-DOX). Recent advances demonstrate that AI is not only a complementary tool but an active driver in the rational design of internal stimuli-responsive nanocarriers. In pH-responsive systems, ML models have been trained on experimental datasets to predict drug release profiles as a function of polymer composition, pKa of ionizable groups, and environmental pH, enabling the optimization of acid-labile linkers and polymer ratios for tumor-specific release behavior. For example, supervised learning algorithms have successfully correlated structural parameters of polymeric micelles with their pH-triggered dissociation and drug release kinetics, significantly reducing experimental trial-and-error [42,43]. Yüceer et al. [42] employed AI techniques, specifically artificial neural networks (ANNs), to simulate the release behavior of doxorubicin from pH- and temperature-sensitive poly(NIPAAm-co-AAc)-PEG IPN hydrogels. His research revealed that the predictions made by the ANN model were found to be more accurate than those obtained by other regression techniques. This technique was useful to efficiently assess the release behavior by varying the pH and temperature. This is because the release behavior was accurately predicted by the ANN model based on specific formulation variables and environmental factors. In another research, the same research group examined the pH- and temperature-dependent mechanical properties of these IPN hydrogels. An inverse correlation was found between swelling and compressive strengths. In addition, the deswelling behavior was predicted by the ANN and least squares-support vector machine (LS-SVM) models. Among these, the ANN model was found to have an exceptionally good correlation with the experimental results. This is due to the ability of the ANN model to accurately simulate the complex deswelling behavior of the IPN hydrogels [42]. The pH-responsive nanomicelles of Pluronic F-127 were formulated for the delivery of oxaliplatin by Fathi-Karkan et al. [44]. They applied ML for the quantitative analysis of the drug release pattern. Improved drug release in acidic conditions compared to physiological pH was achieved by the nanocarriers, validating the potential of the nanocarriers for tumor targeting. ML algorithms, including Random Forest and Gradient Boosting, achieved high prediction accuracy in modeling the drug release pattern, while SHAP analysis helped in understanding the major transitions in the drug release mechanism [44]. Pourmadabi et al. [45] also reported a CMC/agarose/CeO_2_-based nanocomposite for the pH-responsive delivery of quercetin, combined with ML to model drug release behavior. The system showed markedly enhanced release under acidic tumor-like conditions compared to physiological pH, along with selective cytotoxicity toward HepG2 cancer cells while sparing normal cells. ML approaches, including Gradient Boosting, Random Forest, SVM, and neural networks, accurately captured the non-linear pH-dependent release profiles with high predictive performance. This study further demonstrates how ML can support the optimization and precise interpretation of complex release kinetics in pH-sensitive nanocarrier systems [45]. These works collectively show the power of AI in transforming the design of pH-responsive systems from empirical optimization to predictive engineering, in which the polymer and environmental conditions can be pre-selected to enable desired release behaviors with minimal experiment iteration. AI is able to formulate polymers with specific pKa or charge sites, to forecast the effects of polymer constitution on swelling behavior in response to changes in pH or dissociation of micelles, and to propose the ratio of copolymers responding to multiple stimuli. The input values usually involve characteristics of the polymer structure (i.e., the ratio of monomers, pKa, hydrophobic segment size, cross-linking), while the output includes desired polymer compositions, LCST/UCST, or release rates [46]. These models have been employed to optimize block sizes in copolymers and acid-sensitive linkages to ensure maximum loading and sharp release profiles at tumor pH levels [45]. The ANN showed great promise in terms of its ability to learn and predict highly non-linear relationships among the composition of polymers, pH, and the characteristics of drug release from complex delivery systems [[47], [48], [49]]. On the other hand, the ensemble tree models of random forest and gradient boosting showed great promise when dealing with multiple variables and were more interpretable through the use of feature importance analysis [44,48]. In one study, SHAP analysis for a pH-dependent release model revealed that time and pH were the most important predictors, which explained the mechanism of drug release [44]. The support vector machine (SVM) also showed promising results in terms of modeling non-linear trends, especially when there was only a small amount of data available [48].

**Redox-responsive systems:** redox-responsive nanostructures in drug delivery systems are advanced materials engineered to release their therapeutic payload in response to changes in the body's redox environment [50]. These systems exploit the notable differences in redox potential between healthy physiological conditions and pathological sites, such as tumors or inflamed tissues. Pathological environments, including tumor microenvironments and intracellular compartments like the cytoplasm or nucleus, are characterized by elevated concentrations of reducing agents, such as glutathione (GSH), compared to the extracellular milieu. This intrinsic redox gradient serves as a natural trigger for selective and controlled drug release, enhancing therapeutic efficacy while minimizing off-target effects [50,51]. Chen and coworkers [52] developed an innovative stimuli-responsive nanocarrier, comprising a polysaccharide-enveloped liposome constructed using layer-by-layer deposition of redox-responsive amphiphilic chitosan (CS) and hyaluronic acid (HA). This system, termed HCLR, was loaded with survivin-shRNA (an IAP inhibitor) and hyaluronidase (HAase) as a permeation promoter [52]. The HA coating provided stability in blood circulation by maintaining a negative surface charge, while fluorescence resonance energy transfer and in vivo biodistribution studies confirmed the enhanced tumor targeting and accumulation of HCLR. Suppressing survivin expression, significantly suppresses tumor growth with minimal toxicity in mice. This study highlights the potential of HCLR as a promising candidate for clinical translation, owing to its stimuli-triggered tumor targeting, penetration, cellular uptake, and gene release [52]. Lu et al. [53]developed a dual-responsive drug delivery system based on HA and mesoporous silica nanoparticles (MSNs) for cancer therapy. To prevent side reactions between the anticancer drug DOX hydrochloride and HA, the drug delivery system, named DOX@MSN-SS-N=C-HA, utilized host-guest interactions. The MSNs, serving as “nanocontainers,” were functionalized with benzene rings through a combination of pH-responsive benzoic imine bonds and redox-responsive disulfide linkages [53]. Rezaei et al. [54] designed and developed innovative nanoparticles based on hyaluronic acid, chitosan, and lipoic acid (HACSLA-NPs), which are responsive to reduction conditions for the effective treatment of breast cancer. These nanoparticles were designed to target cells overexpressing the CD44 receptor and to enable reduction-triggered release of 17α-methyltestosterone [54]. The results demonstrated that MT/HACSLA-NPs exhibited sustained drug release in the absence of GSH, whereas the presence of GSH resulted in rapid MT release [54]. Other AI-based techniques have been utilized to design redox-sensitive nanocarriers. For example, data-based models have been developed to forecast the stability and degradation rate of disulfide bonds and the release profile mediated by glutathione (GSH) based on their chemical structure and environmental factors [55,56]. DL models, in particular, have demonstrated promise in the prediction of nonlinear relationships between crosslink density, redox sensitivity, and release efficiency. The predictive capabilities of such models allow for the design of responsive carriers with optimized sensitivity to the reducing intracellular environment and stability in the systemic circulation [55,56]. Gorish et al. [57] developed a multi-stimuli responsive nanocomposite formulation (Lignin@GO@ZIF-8) for prostate cancer therapy, which can deliver 5-fluorouracil and metformin while providing pH and redox-mediated drug release. To optimize this formulation, a ML method based on a dataset containing 500 formulation-property pairs was used. From all models used for prediction, XGBoost showed excellent predictive ability for important parameters such as drug loading, encapsulation efficiency, and release profiles (R^2^ ∼ 0.86-0.89). The most important parameters were identified as ZIF-8 content, GO content, size, zeta potential, pH, and glutathione concentrations. This optimization method resulted in improved drug loading efficiency and release profiles compared to non-optimized ones. Moreover, in vitro studies confirmed sustained drug release and excellent therapeutic effects on LNCaP prostate cancer cells while reducing normal cell viability. This work shows the potential for ML methods to optimize nanocomposites for cancer therapy [57]. These approaches underscore the potential of AI in terms of its capability for the simultaneous optimization of stability and responsiveness, thus addressing a major limitation of conventional approaches. The emergence of AI in redox-responsive systems can take the form of optimization of disulfide-thioketal ratios or the design of carriers. Features for input can include the ratio of disulfide-thioketal bonds, hydrophilicity of polymers, and local concentrations of reducing agents; output can involve prediction of cleavage rate and degradation rate and redox-dependent release of drugs. Even though ML applications are still scarce, one can assume by analogy with pH systems that ML will help evaluate the sensitivity of polymeric nanoparticles to GSH [58]. AI can also incorporate multifactor carrier systems (such as disulfide bonds with pH-responsive motifs) to achieve an optimal response. The capability of AI to associate material attributes with release properties also means that AI is able to optimize redox-responsive linkers and materials [46,59].

**Enzymes-responsive systems:** Enzyme-responsive drug delivery systems are innovative platforms specifically designed to harness enzymatic activity in disease-specific environments. These systems are engineered to respond to specific enzymes in the body and release drugs in a targeted manner at the desired locations [60]. In particular, enzymes such as proteases, lipases, and glycosidases are abnormally active in cancerous tissues, inflamed areas, or cells affected by various diseases. These enzymes can break or modify enzyme-responsive linkages in drug delivery systems, leading to the release of the drug at the targeted site [61,62]. The use of specific linkers that are sensitive to particular enzymes allows for more precise drug release at the site of disease, minimizing unnecessary side effects. These systems can also be designed to respond to multiple enzymes in more complex conditions, such as in cancer therapy, where several types of enzymes are involved in different stages of the disease [60,63]. Ultimately, by utilizing the specific characteristics of enzymes, the enzyme-responsive systems can significantly enhance the precision and efficacy of drug delivery, providing more targeted and accurate treatments for diseases [63]. Alkekhia and coworkers [64] developed hydrogels that degrade specifically in the presence of β-lactamases and β-lactamase-producing bacteria, offering a bacteria-triggered drug delivery platform. They utilized a maleimide-functionalized β-lactamase-cleavable cephalosporin as a crosslinker to fabricate the hydrogels through end-crosslinked polymerization with thiol-terminated poly (ethylene glycol). In vitro and ex vivo testing showed that only the hydrogels containing the responsive crosslinker were degraded by β-lactamases and β-lactamase-producing bacteria. Guo et al. [65]developed enzyme-responsive nanoparticles (Cur-P-NPs) based on a functionalized copolymer (mPEG-Peptide-PCL) sensitive to matrix metalloproteinase (MMP) to improve drug bioavailability and therapeutic targeting in cancer treatments. The in vitro release of curcumin (Cur) from Cur-P-NPs was stable in the acidic tumor micro-environment, ensuring consistent therapeutic effects [65]. In enzyme-responsive systems, AI has been used to optimize the specificity and efficiency of enzyme-degradable linkers [66]. Computational approaches, including ML-based peptide screening and molecular docking integrated with AI, allow the identification of enzyme-sensitive sequences with high selectivity toward disease-associated enzymes such as matrix metalloproteinases (MMPs). Furthermore, predictive models can estimate degradation rates and drug release kinetics based on enzyme concentration and substrate affinity, facilitating the development of highly targeted and controllable delivery systems [66,67]. Zimmerman et al. [68] proposed a strategy for enzyme engineering using the power of DL to design highly expressed, thermally stable, and catalytically active enzymes without the need for extensive experimental screening. The strategy was named CoSaNN, which stands for Conformation Sampling using Neural Networks. The proposed strategy uses context-aware modeling to examine the complex and non-linear relationship between the sequences and structures of proteins. This strategy helps in the modulation of the conformation of the enzyme. The researchers simultaneously proposed the use of the graph neural network model named SolvIT to predict the solubility of the proteins in *Escherichia coli*. The proposed strategy was successful in designing a significant proportion of the target proteins with increased expression levels and thermal stability compared to the wild-type template. The proposed strategy was successful in maintaining the functional activity of the target proteins. The proposed strategy shows the potential of AI in capturing the structural interactions in proteins and maintaining the allosteric properties of the target proteins. The proposed strategy shows the potential of AI in streamlining the enzyme engineering process [68]. These advances suggest that AI not only improves enzyme engineering but also directly enhances the precision of enzyme-responsive nanocarriers by allowing the rational selection of the linker sequences with well-characterized cleavage behavior in complex biological environments. Table 2 provides a comprehensive overview of internal-stimuli responsive nanocarriers, including those triggered by pH changes, redox conditions, and enzymatic activity. These nanocarriers represent an advanced approach in controlled drug release systems, with the ability to respond to specific micro environmental conditions in pathological tissues. In the pH-responsive category, various materials such as nanoparticles [89,92], liposomes [86,87], metal-organic frameworks (MOFs) [75,90], micelles [88], and hydrogels [91] are utilized. These systems are activated by pH variations in tumor environments or intracellular organelles (endosomes and lysosomes), leading to the selective release of therapeutic agents. For instance, acetalated dextran nanoparticles [69] delivering platinum and zinc oxide nanoparticles [70] transporting quercetin to breast cancer cells are notable examples in this category. In the redox-responsive section, these nanocarriers exploit the distinctive redox characteristics of tumor environments, particularly the elevated levels of reducing agents such as glutathione. This category includes nanoparticles [106,107] and redox-responsive hydrogels [98] that release their cargo by breaking disulfide bonds or other redox-responsive linkages in the presence of reducing agents. Prominent examples include polyurethane [97] and hyaluronic acid-based nanoparticles [107] for delivering doxorubicin to breast cancer cells and gold nanoparticles coated with hyaluronic acid and folic acid [107] for targeting breast cancer and HeLa cells. In the enzyme-responsive category, these nanocarriers rely on the presence of specific enzymes overexpressed in pathological tissues, such as tumors or inflamed areas, to trigger drug release. Examples include mesoporous silica nanoparticles responsive to hyaluronidase, which release 5-fluorouracil for colon cancer treatment [108], and alginate and poly-lysine nanoparticles for the controlled delivery of ciprofloxacin [112]. These smart delivery systems not only enhance selectivity and minimize side effects but also offer multi-responsive capabilities, allowing precise control over drug release depending on the biological environment. The role of AI in designing enzyme sensitive linkers and coatings is done by considering the peptides or degradable components as inputs. Features used could be the enzyme concentration, peptide substrate sequence or its cleavage rate constant, the polymer composition, and particle size while output is predicted degree of cleavage or onset of shape change/release. For example, cleavable peptide sequences by matrix metalloproteases are embedded into hydrogels or nanoparticles [121]. AI can also be employed to identify or evolve these peptides with the aim of maximizing specificity and efficiency. According to a study, it was demonstrated that MMP-cleavable peptides incorporated within nanocarriers resulted in local gelation or delivery of cargo [121] and ML would enhance this process through identification of correlations between the peptide sequence and cleavage results. Thus, examples of AI applications in enzyme-controlled systems are peptide prediction and cargo delivery kinetic modeling [121,122]. The conclusion drawn from Table 2 indicates that internal-stimuli responsive nanocarriers provide an innovative and effective platform for improving disease treatment, particularly cancer therapy. Each of these systems, depending on the type of stimulus, employs distinct mechanisms to trigger drug release, leading to enhanced therapeutic efficacy, reduced side effects, and improved target specificity. Future research in this field could focus on the development of multi-responsive smart nanocarriers, improving biostability, and integrating advanced technologies such as AI for optimized design. Ultimately, these advancements could pave the way for more personalized and precise therapies, significantly enhancing patients’ treatment outcomes. Concurrent with the progress in material chemistry, AI has started to assume a growing role in the design and optimization of internal stimuli-responsive nanocarriers [123,124]. ML and DL models have been used to predict the structure-property relationships underlying pH sensitivity, redox-triggered bond cleavage kinetics, and enzyme-responsive degradation profiles [125]. Through learning from experimental data sets that correlate formulation parameters (such as polymer composition, linker chemistry, and crosslink density) with the behavior of drug release and biodistribution results, AI models can uncover complex nonlinear relationships that are hard to discern using traditional empirical methods. In pH-responsive formulations, predictive models help to optimize acid-labile linker composition and ionizable polymer ratios [125]. In redox-responsive platforms, data-driven strategies allow the prediction of the stability of disulfide bonds and the dynamics of glutathione-mediated release. Likewise, in enzyme-responsive platforms, computational modeling can facilitate the choice of enzyme-cleavable peptides with higher specificity and controlled degradation rates [123,124].

**Table 2:** Internal-stimuli responsive nanocarriers.

Year/Ref	Cancer/Cell Type	Therapeutic Compound	Component	Nanocarrier	Internal Stimuli
2019/[69]	Cancer cells	Platinum	Acetalated dextran	Nanoparticles	pH pH
2019/[70]	Breast cancer	Quercetin	Zinc oxide	Nanoparticles	
2020/[71]	Cervical cancer	Carboplatin, Paclitaxel	PEG^b^	Lipid-polymer hybrid nanoparticles	
2020/[72]	Hepatocellular Carcinoma	Sorafenib, Curcumin	Lactobionic acid	Nanoparticles	
2020/[73]	DNA vaccines	-	SS-cleavable and pH-activated lipid-like material and vitamin E	Liposome	
2020/[74]	-	Doxorubicin	PLGA a	Polymeric Nanoparticle	
2020/[75]	glioblastoma	5-fluorouracil	Hyaluronic acid	Metal-organic framework	
2020/[76]	Hela Cells	Doxorubicin	tripeptide Lys-Phe-Gly (KFG)	Gold nanoparticle	
2020/[77]	solid tumors	Doxorubicin	PEG, and folate-conjugated graphene oxide	Gold nanoparticle	
2020/[78]	Liver cancer	Quercetin	PEG_5k_-PAE_10k_	Nanodots	
2020/[79]	-	Doxorubicin	Fe3O4@C@TDGQDs microspheres	Maltose-functionalized dendrimer/graphene quantum dots	
2020/[80]	MCF-7, HCT-116 cells	Doxorubicin	poly(ethylene glycol)-b-polycarbonate-b-oligo([R]-3-hydroxybutyrate)	Micelles	
2020/[81]	HeLa Cells	Doxorubicin	polyamide-amine (PAMAM)	Dendrimer	
2020/[82]	-	Amphiphilic drugs	PEG, Chitosan	Micelles	
2021/[83]	Breast cancer	Methotrexate	sodium borohydride, ascorbic acid, citrate acid monohydrate, hydrochloric acid	Gold/gelatin nanoparticle	
2021/[84]	Breast cancer	Doxorubicin	Acid-responsive peptide (DVar7)	Liposomes	
2021/[85]	MCF-7 and L929	Curcumin	Citraconic anhydride (CA), DSPC, DSPE-PEG2000	Liposomes	
2021/[86]	HBMEC and C6	Calcium arsenite	Egg phosphatidylcholine, CHOL, DSPE-PEG2000- angiopep-2	Liposomes	
2021/[87]	HEK-293 and GL261	TRAM-34	EPC, PDMAEMA-b-PLMA diblock copolymer	Liposomes	
2021/[88]	SW620 and DU145 cells	Paclitaxel (PTX) and Nile Red dye	PEG- blockpoly(cyclohexyloxy ethyl glycidyl ether)s	Micelles	
2022/[89]	Breast cancer	Doxorubicin, vincristine	Chitosan	Nanoparticle	
2022/[90]	Breast cancer	6-Mercaptopurine	Chitosan	Metal-organic framework	
2024/[91]	Breast cancer	Doxorubicin	starch/PVA/g-C_3_N_4_	Hydrogel	
2024/[92]	MCF-10 A and cancer MCF-7 cells	Methotrexate	Chitosan	Nanoparticle	
2024/[93]	-	Taxol	Acetalated Dextran_6k_-PEG_5k_-PLA_2k_	Nanoparticle	
2018/[94]	-	Curcumin	4,4′-dithiobisbenzoic acid (4,4′-DTBA)	Metal-organic framework	Redox
2021/[95]	-	Ribonuclease A	Sialic Acid, Disulfide Bonds, GSH	Nanoparticle	
2021/[96]	Breast Cancer	siRNAs	PEG, polyethylenimine	Nanoparticle	
2022/[97]	Breast cancer	Doxorubicin	Polyurethane, hyaluronic acid	Micelles	
2022/[98]	colorectal cancer	-	manganese oxide (MnO_2_)	Hydrogel	
2022/[99]	colorectal cancer	Doxorubicin, hydrophobic curcumin	poly(allylamine)/eudragit S-100	Nanoparticle	
2023/[100]	Myocardial infarction	PF543	DSPE-PEG	Nanoparticle	
2023/[101]	Breast Cancer	Doxorubicin	Bis (2-hydroxyethyl) disulfide	Copolymer nanoparticle	
2023/[102]	-	Doxorubicin	trithiocyanuric acid	Nanoparticle	
2024/[103]	Breast Cancer	Docetaxel	MeO-PEG-b-(NIPAAm-co-PBAE)	Nanoparticles	
2024/[104]	Breast Cancer	Paclitaxel	PEG, disulfide	Nanoparticles	
2024/[105]	-	Mitochondrial	Pep-CS-LND	Hydrogel	
2024/[106]	MDA-MB-231 cell line	Doxorubicin	Chitosan	Nanoparticles	
2024/[107]	HeLa Cells, breast cancer (BT-20) cells, (HEK-293) cells	Methotrexate	Hyaluronic acid, folic acid	Gold nanoparticles	
2018/[108]	Colon cancer	5-fluorouracil	Hyaluronic acid	Mesoporous silica nanoparticles	Enzyme
2018/[109]	Colorectal cancer	Doxorubicin	Poly(ethylene glycol)-b-poly(L-tyrosine)	Nanoparticles	
2018/[110]	-	Oxaliplatin	Pep-Pt-P	Nanoparticles	
2019/[111]	-	Paclitaxel	Saccharide	Polymeric nanoparticle	
2021/[112]	-	Ciprofloxacin	Alginate, poly-l-lysine	Nanoparticles	
2022/[113]	-	Doxorubicin	-	Metal-organic framework	
2023/[114]	Diabetic, inflammatory wounds	Dexamethasone	(N-hexanoic acid)−5-norbornene-exo-2,3-dicarboximide (NorAHA)	Nanoparticles	
2023/[115]	Colorectal cancer	Eugenol	Cetyltrimethylammonium bromide, tetraethyl orthosilicate, (3-aminopropyl) triethoxysilane	Nanoparticles	
2023/[116]	-	Antibacterial agents	Chitosan, polycaprolactone, polyvinyl alcohol	Nanoparticles	
2023/[117]	Breast Cancer	Doxorubicin	chitosan/gelatin hybrid nanogel	Gold nanoparticles	
2023/[118]	HeLa versus MDA-MB-231 and MCF7	Doxorubicin	N_3_–PEG–PO_3_	Metal-organic framework	
2024/[119]	Antibacterial Therapy	Cationic antimicrobials	PEG, poly(ɛ-caprolactone) (PCL) and phosphonic acid-bearing methacrylate	Nanoparticles	
2024/[120]	tumor cells	Doxorubicin	mPEG_79_-GFLGDDD	Copolymer nanoparticle	

In fact, a more detailed analysis of Table 2 demonstrates some major trends in designing responsive nanocarriers according to different internal stimuli. With regard to the types of stimuli, the largest body of research involves pH-responsive delivery systems, which can be explained by the presence of pH gradients between tumor tissue and normal cells, as well as different compartments within a cell. As for the materials used for creating the carrier structures, polymer nanoparticles, liposomes, micelles, and hydrogels dominate among other platforms, mainly due to their flexible physical and chemical properties and biocompatibility. When it comes to the nature of active ingredients, the most commonly used substances include doxorubicin, 5-fluorouracil, platinum drugs, and other anticancer drugs, which shows that the primary area of application for responsive nanocarriers remains chemotherapy. Not surprisingly, a large share of studies aims at delivering drugs to tumors, especially breast, colon, and cervical cancers. Moreover, one can observe a growing tendency towards developing multiple trigger responsive nanocarriers involving two or even three different stimuli (e.g., pH-redox, enzyme-redox), and employing AI techniques becomes increasingly common in the field.

### 2.2 External stimuli

External stimuli-responsive nanostructures have emerged as a transformative innovation in nanotechnology, owing to their remarkable ability to dynamically adapt to environmental cues in a controlled, precise, and often reversible manner. These advanced systems are meticulously engineered to undergo specific and predictable changes in their physical, chemical, or biological properties when exposed to external triggers such as temperature, light, or magnetic fields. This type of responsiveness enables them to act as “smart” systems, capable of delivering tailored functionalities under pre-defined conditions [126,127]. The versatility of these nanostructures lies in their ability to fine-tune their behavior by leveraging unique material properties such as phase transitions, molecular conformational changes, or surface modifications. This dynamic adaptability positions them as a cornerstone in modern biomedical research and industrial applications. Specifically, they hold immense potential in drug delivery systems, where external stimuli can regulate drug release at targeted sites, minimize systemic side effects and maximize therapeutic efficacy [128]. Beyond drug delivery, stimuli-responsive nanostructures play a pivotal role in tissue engineering by creating environments that mimic physiological conditions, thereby enhancing cell growth and tissue repair. Additionally, their integration into diagnostic imaging systems improves sensitivity and specificity, enabling real-time monitoring of disease progression or therapeutic outcomes [129,130].

**Fig. 1:**
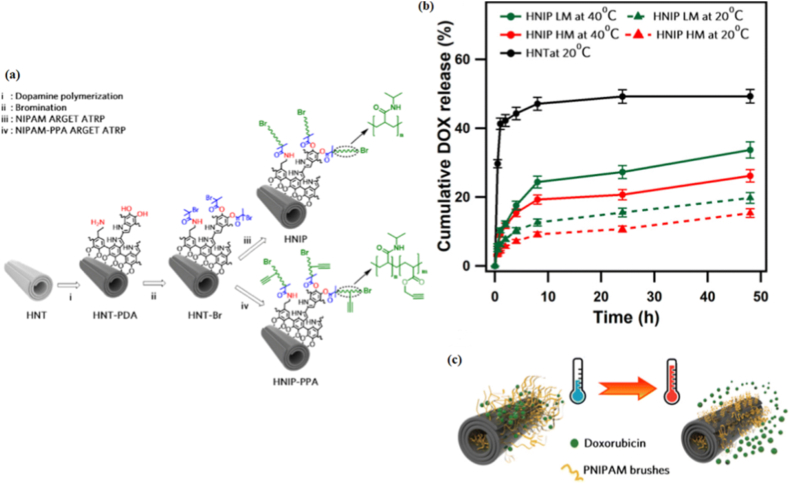
(a) The schematic representation of the synthesis protocol for modifying halloysite nanotubes (HNTs) involves several key steps. First, HNTs are coated with polydopamine (i), providing a surface for further functionalization. Next, alkyl bromide moieties are grafted onto the surface of the polydopamine-modified nanotubes (ii). Finally, PNIPAM brushes (iii) or poly (NIPAM-PPA) brushes (iv) are decorated onto the modified HNTs, completing the synthesis of the temperature-responsive nanocarriers. (b) Cumulative DOX release profiles for pristine and PNIPAM-grafted HNTs at different temperatures. (c) Schematic representation of the temperature-responsive release mechanism of DOX from PNIPAM-grafted HNTs. Reproduced with permission from Ref. [142]. Copyright 2023, Royal Society Chemistry.

**Temperature-responsive systems:** temperature-responsive nanostructures are a specialized class of materials that can dynamically adjust their physical or chemical properties in response to thermal changes. These nanostructures are primarily designed to leverage local or systemic temperature variations, enabling targeted and controlled drug release. The mechanism of action typically relies on thermally induced phase transitions or molecular conformational changes, which are triggered by crossing a predefined temperature threshold [131]. Polymers such as poly(N-isopropylacrylamide) (PNIPAM) are commonly employed due to their unique lower critical solution temperature (LCST) behavior [132]. At temperatures above their LCST, these polymers transition from a hydrated, hydrophilic state to a dehydrated, hydrophobic state, causing structural collapse and subsequent release of encapsulated agents [132,133]. This phenomenon has been exploited in the development of nanocarriers for applications such as hyperthermia-induced drug delivery, where the controlled release of chemotherapeutics is achieved under mild hyperthermic conditions [133]. Thermosensitive liposomes [134], micelles [135], and hydrogels [136] represent key examples of temperature-responsive systems. These platforms not only enable spatially localized delivery but also minimize off-target effects by confining therapeutic release to regions experiencing elevated temperatures. This property is particularly advantageous in cancer treatments, as hyperthermic zones can be induced noninvasively using external sources such as infrared radiation or focused ultrasound. For instance, liposomes modified to release DOX upon heating have shown promising results in preclinical studies, enhancing drug accumulation in tumor tissues while reducing systemic toxicity [137,138]. Another significant contribution of AI tools has been in the optimization of temperature-responsive nanocarriers. The phase transition behavior and the lower critical solution temperature (LCST) of thermoresponsive polymers can be predicted using AI tools. The ML model can be trained on the composition and physicochemical properties of the polymers to predict the LCST of the polymers. This helps in designing systems with the required response in the narrow range of temperatures required for hyperthermia-based treatments. Boztepe et al. [46] explored the application of AI in modeling the drug release from a dual pH and temperature responsive hydrogel system based on poly(N-isopropyl acrylamide-co-acrylic acid)/poly(ethylene glycol) interpenetrating polymer networks. In the study, the hydrogels were prepared by free radical polymerization and showed fast response characteristics in terms of shrinking in response to environmental stimuli. Doxycycline hydrochloride was incorporated into the hydrogels, and its release was investigated under different pH and temperature conditions. To solve the difficulty in modeling the drug release from hydrogels, various AI modeling tools were used in the study, including ANN, LS-SVM, and support vector regression (SVR). Comparison of the modeling results using various statistical parameters like correlation coefficient, RMSE, MSE, and MAPE showed that the drug release from hydrogels can be best modeled by the ANN model. This study clearly demonstrates the potential of AI modeling in the development of hydrogels for drug delivery [46]. AL-Rajabi et al. [139] have successfully employed a hybrid ML approach to forecast the drug release behavior of silver sulfadiazine from temperature-sensitive PF-127-based hydrogels. In this study, various ML models were trained with experimental data obtained from various formulation and temperature conditions. Among the ML models employed, the Random Forest approach was found to have the highest prediction accuracy. Not only was the ML approach successful in understanding the impact of temperature, polymer concentration, and drug content on drug release, but it was also found to be consistent with zero-order kinetics, the Higuchi model, and non-Fickian diffusion [139]. Mahdian et al. [140] developed a dual stimuli-responsive hydrogel for pH- and temperature-responsive delivery of curcumin (CUR). The hydrogel, stable at 37 °C, enhanced CUR release in acidic conditions and under near-infrared (NIR) irradiation. CUR release at pH 5.0 with NIR was 1.43 times higher than at pH 7.4, demonstrating its potential for targeted cancer therapy [140]. Gao and coworkers [141] synthesized degradable magnetic nanogel composites (PNVCL/Fe_3_O_4_ NCGs) with dual temperature- and redox-responsiveness using inverse mini-emulsion polymerization. These nanogels, incorporating vinyl-modified Fe_3_O_4_ nanoparticles, attached chemically to a polymer matrix, and demonstrated excellent structural integrity in aqueous environments [141]. The NCGs exhibited superparamagnetic properties, reversible temperature responsiveness, and redox sensitivity. Controlled release of anticancer drug 5-fluorouracil was achieved by tuning either one of external temperature and redox conditions, or both, while maintaining low cytotoxicity, making them promising candidates for guided drug delivery [141]. AI techniques are employed for optimization of thermal transitions (LCST) and thermally induced drug delivery systems. Input parameters include polymer characteristics (monomer identity, molecular mass, crystallinity, block copolymer composition) and temperature profile, whereas output parameters include transition temperature, swelling ratio, and release kinetics. Neural networks predicted doxorubicin release from PNIPAAm-PAAc hydrogels at various pH and temperature conditions effectively [46]. AI is able to come up with copolymer formulae in order to obtain the desired LCST or cause immediate collapse above a certain temperature level. When using photothermal triggers, AI is able to optimize the number of particles as well as the duration for which the irradiation lasts so as to heat the system without causing any harm [46,59]. Hemmatpour et al. [142] developed a temperature-responsive nanocarrier based on halloysite nanotubes (HNTs), a biocompatible clay mineral, for controlled anticancer drug release (Fig. 1a). As shown in Fig. 1a, the HNTs were first modified with a polydopamine layer, followed by Poly(N-isopropylacrylamide) (PNIPAM) brush grafting through atom transfer radical polymerization. The introduction of PNIPAM, a thermoresponsive polymer with an LCST of about 32 °C, allows for reversible structural changes in response to temperature fluctuations. It is also important to note that the increased polymerization degree of PNIPAM brushes increased the drug-loading capacity (up to 250 mg g^−1^ for HNIP HM compared to 160 mg g^−1^ for HNIP LM), which underlines the role of hydrogen bonding interactions between DOX and PNIPAM chains. The PNIPAM brushes reduced cytotoxicity and enabled temperature-dependent drug release, demonstrated with doxorubicin-loaded nanotubes. STED nanoscopy confirmed the internalization of the nanotubes into HeLa cells, highlighting their potential in hyperthermia-based cancer therapies [142]. The temperature-dependent drug release behavior is quantitatively presented in Fig. 1b. Pristine HNTs exhibited a pronounced burst release, with nearly 40% of the loaded DOX released within the first hour, primarily due to electrostatic adsorption of drug molecules on the outer nanotube surface. In contrast, PNIPAM-grafted samples (HNIP HM and HNIP LM) demonstrated a gradual and sustained release profile without an initial burst effect. This behavior confirms the role of the PNIPAM shell as a diffusion barrier that restricts rapid drug escape. Furthermore, as shown in Fig. 1b, temperature significantly influenced release kinetics: at 40 °C (above LCST), approximately 25% of DOX was released within 8 h, whereas only 15% was released at 20 °C (below LCST) [142]. The underlying mechanism is schematically depicted in Fig. 1c. Below the LCST (20 °C), PNIPAM chains remain in a hydrated and extended conformation due to hydrogen bonding with surrounding water molecules, stabilizing DOX through polymer–drug interactions and limiting diffusion. Above the LCST (40 °C), hydrogen bonds are disrupted, leading to polymer chain collapse and shrinkage, which reduces steric confinement and facilitates enhanced drug release. Moreover, the thicker PNIPAM layer in HNIP HM provided a stronger diffusion barrier compared to HNIP LM, resulting in slightly slower release kinetics. These findings verify that PNIPAM brushes control drug release based on temperature changes, thus suppressing burst release and achieving controlled release. In summary, Fig. 1 illustrates how polymer structure and temperature-triggered conformation changes work together to control drug loading and release, representing an important design concept for temperature-sensitive nanocarriers in hyperthermia-based chemotherapy [142].

**Fig. 2:**
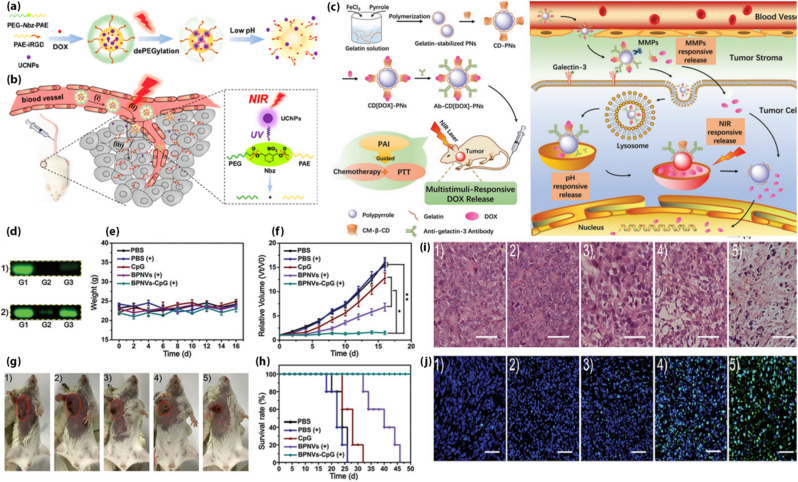
(a) Schematic representation of nanodrugs responsive to NIR light and pH changes. (b) Diagram depicting NIR-guided nanodrugs overcoming physiological barriers, including (i) blood circulation, (ii) extravasation, and (iii) tumor infiltration. Reprinted with permission from Ref. [152]. Copyright 2019, American Chemical Society. (c) Diagram depicting the design, composition, and multifunctional properties of polypyrrole nanoparticles responsive to multiple stimuli. Reprinted with permission from Ref. [153]. Copyright 2019, John Wiley and Sons. In Vivo Cancer Treatment Evaluation. (d) Agarose gel electrophoresis results: 1) Lane G1: CpG alone, G2: BPNVs, and G3: BPNVs-CpG; 2) Lane G1: CpG alone, G2: BPNVs-CpG, and G3: BPNVs-CpG exposed to NIR laser irradiation. (e) Body weight measurements of mice across various treatment groups. (f) Tumor growth trends over time. (g) Representative images of mice from different treatment groups on day 16. (h) Survival rates of mice subjected to different treatments. (i) H&E-stained tumor tissue sections from each treatment group (Scale bar = 50 μm). (j) TUNEL-stained tumor images, with scale bar = 50 μm (blue: DAPI, green: TUNEL-FITC). ∗p < 0.05, ∗∗p < 0.01. (+) denotes NIR laser exposure (150 mW cm^−2^) for 10 min. Reprinted with permission from Ref. [154]. Copyright 2019, John Wiley and Sons. (For interpretation of the references to color in this figure legend, the reader is referred to the Web version of this article.)

**Light-responsive systems:** Light-responsive nanostructures represent a cutting-edge approach to achieving precise spatiotemporal control over drug release in controlled release systems [143]. These systems are engineered with photo-responsive materials, such as photochromic compounds, light-responsive polymers, or photosensitizers, which undergo structural or chemical transformations upon exposure to specific wavelengths of light [144]. The interaction with light, including ultraviolet (UV), visible, or NIR radiation, can trigger processes such as bond cleavage, conformational changes, or heat generation, enabling targeted release of encapsulated therapeutic agents. NIR light, in particular, is preferred due to its deeper tissue penetration and minimal phototoxicity impact on surrounding healthy tissues [145]. Recent advances have demonstrated the use of nanostructures such as gold nanoparticles, quantum dots, and up-conversion nanoparticles to enhance the photothermal or photodynamic effects, enabling synergistic therapeutic outcomes. For example, light-responsive micelles loaded with photosensitizers have been shown to generate reactive oxygen species under light activation, effectively disrupting cancer cells while minimizing systemic toxicity [146]. This versatility positions light-responsive nanostructures as promising candidates for applications ranging from targeted drug delivery to theranostics, advancing the frontiers of precision medicine [143]. Chen and coworkers [147] developed red-light-responsive metallopolymer nanocarriers (DOX@PolyRuCHL micelles) to treat multidrug-resistant tumors. These nanocarriers deliver DOX and chlorambucil (CHL) to resistant cancer cells and release them upon red light irradiation. The system effectively overcomes drug resistance by enabling cellular uptake of DOX and triggering synergistic anticancer effects [147]. The approach demonstrated significant tumor inhibition in a mouse model, offering a promising strategy for photoactivated chemotherapy [147]. He and coworkers [148] developed an innovative “all-functions-in-one” nanocarrier system for targeted protein delivery and precise control of protein activity using low-power, long-wavelength visible light (635 nm). The system combines reactive oxygen species (ROS)-responsive protein engineering and photosensitizer-mediated ROS generation [148]. RNase A was modified with an H_2_O_2_-cleavable phenylboronic acid derivative (RNBC) and encapsulated in acid-degradable, ketal-crosslinked PEI (KPEI)-based nanocomplexes coated with PS-modified hyaluronic acid (HA). The HA coating ensures tumor targeting and prolonged circulation, while KPEI facilitates endosomal escape and intracellular release of RNBC. ROS are generated upon light irradiation to activate RNBC and restore protein function, achieving synergistic anti-cancer effects [148]. Light-guided nanovehicles have emerged as a promising approach for active drug delivery within physiological environments. Light offers additional control over light-responsive materials through adjustments in intensity, frequency, polarization, and direction, enabling precise spatial and temporal manipulation [149]. Materials responsive to light, such as photoelectrochemical cells and photothermal agents, can harness photon energy in biological settings without the need for chemical fuel, generating electrochemical or thermal gradients. For example, asymmetric photocatalytic heterostructures such as Au/TiO2, Au/Fe2O3, and Au/Cu2O can produce self-electroosmosis flow by separating electron-hole pairs under low concentrations of hydroxy peroxide and low-power UV light [150]. This knowledge of photocatalytic heterostructures is already being utilized to enhance photocatalytic efficiency and can be readily adapted for the development of light-guided nanovehicles [150]. For light-responsive nanocarriers, the application of AI has been focused on the optimization of the photophysical properties such as the efficiency of light absorption, the conversion rate to heat, and the production of ROS. ML algorithms have been used to predict the behavior of photosensitizers and nanomaterials when exposed to different wavelengths and intensities of light. This has facilitated the rational choice of the material for deep tissue penetration and low levels of phototoxicity. Furthermore, the application of AI has been used to simulate the design of nanocarriers with controlled release kinetics. Hsiao et al. [151] introduced an advanced multimodal therapy platform utilizing light-driven AI-based micro/nanorobots for the treatment of bladder cancer. The platform consists of phototactic and photosynthetic Chlamydomonas reinhardtii in combination with glycol chitosan-polypyrrole nanoparticles. The system enables the synergistic effects of phototherapy and immune modulation. The biohybrid construct not only helps in the precise targeting and therapy of the tumor but also helps in the alleviation of tumor hypoxia by the photosynthetic production of oxygen. Moreover, the approach has the advantage of modifying the immune microenvironment in the tumor by stimulating cytotoxic T cell responses and regulating macrophage polarization. The in vivo studies showed significant inhibition of tumor growth and the induction of immune memory with low chances of recurrence in mice. The application of AI in the design of bioinspired responsive systems has the potential to create precise and immunologically active cancer therapies [151]. ML supports in developing smart carriers based on light irradiation such as photochemical bond cleavage and photothermal properties of particles. The input features comprise of chromophoric absorption spectrum, intensity of light wavelength, composition of particles (for example MoS_2_ or Au), as well as the glass transition of polymers. The output includes the optimum light irradiation conditions, release time of drug molecules, photothermal temperature elevation, or ROS generation. Interestingly enough, ML was used in optimizing NIR laser irradiation of MoS_2_-polymers microparticles [58]. This indicates the capability of AI to combine light parameters and physical characteristics for optimizing the therapy's effectiveness. Overall, ML algorithms may be applied to establish a connection between the optical properties of nanoparticles and light exposure and determine the release efficiency thereof [58]. Zhou et al. [152] proposed a light-triggered PEGylation/dePEGylation strategy to overcome the classical trade-off between prolonged circulation and deep tumor penetration of nanocarriers. As schematically illustrated in Fig. 2a, the system consists of two amphiphilic polymers: PEG-Nbz-PAE-Nbz-PEG (HTMP), in which PEG is linked through photocleavable o-nitrobenzyl (Nbz) groups to a pH-responsive poly(β-aminoester) (PAE) core, and iRGD-PAE-iRGD (iPHT), serving as the targeting component. Core–shell NaYF4:Yb/Tm@NaYF4 up-conversion nanoparticles (UCNPs) are embedded within the nanostructure to convert externally applied near-infrared (NIR) light into localized UV–visible emission. Under physiological conditions, the PEG shell ensures colloidal stability and prolonged blood circulation. Upon NIR irradiation at the tumor site, however, the UCNPs generate UV light that cleaves the Nbz linkers, leading to dePEGylation and exposure of iRGD ligands. This light-triggered structural transformation represents the central design principle illustrated in Fig. 2a [152]. Fig. 2b further demonstrates the biological consequence of this NIR-induced dePEGylation. Removal of the PEG corona reduces steric shielding and activates iRGD-mediated tumor targeting, thereby enhancing vascular extravasation and deep tumor penetration. The dual NIR/pH responsiveness enables spatiotemporal control over PEG detachment specifically within the tumor microenvironment, resulting in improved intratumoral distribution and enhanced antitumor efficacy [152]. Ma and colleagues [153] developed a multifunctional nanoplatform for the treatment of refractory thyroid cancer that integrates photothermal therapy, imaging capability, and triple-stimuli-responsive drug release. As illustrated in Fig. 2c, the nanostructure is centered on gelatin-stabilized polypyrrole nanoparticles (PNs), which serve as both photothermal agents and photoacoustic imaging (PAI) contrast enhancers. Unlike conventional PNs stabilized with synthetic polymers such as PVP or PVA, gelatin was employed to improve biocompatibility and provide abundant functional groups for surface modification. Carboxymethyl-β-cyclodextrin (CM-β-CD) was conjugated to the gelatin shell, enabling supramolecular loading of doxorubicin (DOX) through host–guest interactions [153]. Fig. 2c further highlights the multi-level responsiveness of the system. Drug release is triggered by three complementary stimuli within the tumor microenvironment: (i) enzymatic degradation of gelatin, (ii) acidic pH conditions, and (iii) photothermal heating generated by polypyrrole under near-infrared irradiation. In addition, anti-galectin-3 antibodies were conjugated to the nanoparticle surface to exploit the overexpression of galectin-3 in differentiated thyroid carcinoma. This active targeting strategy enhanced tumor accumulation and significantly increased intracellular DOX uptake via clathrin-mediated endocytosis, as depicted in the figure. By combining passive targeting, antibody-mediated active targeting, and externally triggered photothermal effects, the platform achieved enhanced tumor specificity while reducing systemic cardiotoxicity [153]. The complete tumor eradication without recurrence demonstrated in Fig. 2c underscores the therapeutic advantage of integrating imaging guidance, controlled drug release, and targeted chemo-photothermal therapy within a single nanostructure. The results showed complete tumor eradication without recurrence, highlighting this approach as a promising strategy for thyroid cancer treatment (Fig. 2c) [153]. Li et al. [154] engineered near-infrared/reactive oxygen species (NIR/ROS)-responsive black phosphorus quantum dot vesicles (BPNVs) for synergistic photodynamic immunotherapy. The vesicles were constructed through the self-assembly of amphiphilic BPQDs grafted with polyethylene glycol (PEG) and ROS-responsive poly(propylene sulfide) (PPS), forming hollow nanostructures capable of encapsulating immunoadjuvant CpG oligodeoxynucleotides (CpG ODNs). Upon NIR irradiation, BPNVs generate elevated levels of ROS, which oxidize hydrophobic PPS into hydrophilic segments, thereby inducing vesicle disassembly and releasing both small BPQDs and CpG for deep tumor penetration and immune activation [154]. Fig. 2d and e experimentally validate the loading efficiency and NIR-triggered release behavior of CpG within the vesicles. As shown in Fig. 2d-1, agarose gel electrophoresis revealed negligible CpG leakage from BPNVs-CpG (lane G3) compared to free CpG (lane G1), confirming efficient encapsulation within the hollow cavity stabilized by the hydrophobic PPS layer. In contrast, after NIR laser irradiation (Fig. 2d-), a marked increase in CpG migration was observed, demonstrating on-demand release triggered by vesicle disassembly. Fig. 2e further confirms the in vivo safety of the platform, as no significant body weight loss was detected across treatment groups during the 16-day therapeutic period [154]. The therapeutic efficacy is systematically presented in Fig. 2(f–h). As shown in Fig. 2f and g, mice treated with free CpG exhibited minimal tumor inhibition, likely due to rapid systemic clearance and poor cellular uptake. Similarly, BPNVs with NIR irradiation induced only moderate tumor suppression attributable to photodynamic therapy (PDT) alone. In contrast, the BPNVs-CpG + NIR group displayed the most pronounced tumor growth inhibition, reflecting the synergistic combination of ROS-mediated PDT and CpG-enhanced immunotherapy. Fig. 2h further demonstrates the survival advantage of this combinational approach, with treated mice surviving beyond 50 days, significantly outperforming all other groups [154]. Histological analyses shown in Fig. 2i and j provide mechanistic confirmation at the tissue level. Hematoxylin and eosin (H&E) staining (Fig. 2i) reveals extensive tumor cell shrinkage and chromatin condensation in the BPNVs-CpG + NIR group, indicative of severe tumor damage. Correspondingly, TUNEL staining (Fig. 2j) shows the highest percentage of apoptotic cells in this group, verifying enhanced tumor cell apoptosis. Collectively, Fig. 2d–j illustrate the complete mechanistic cascade of this platform: efficient CpG encapsulation, NIR-triggered ROS-mediated vesicle disassembly, controlled immunoadjuvant release, synergistic photodynamic–immunotherapeutic tumor suppression, and prolonged survival. This integrated evidence highlights a key design principle of stimuli-responsive BP-based nanocarriers—dynamic structural transformation under external stimulation to achieve spatiotemporally controlled combination therapy [154].

**Fig. 3:**
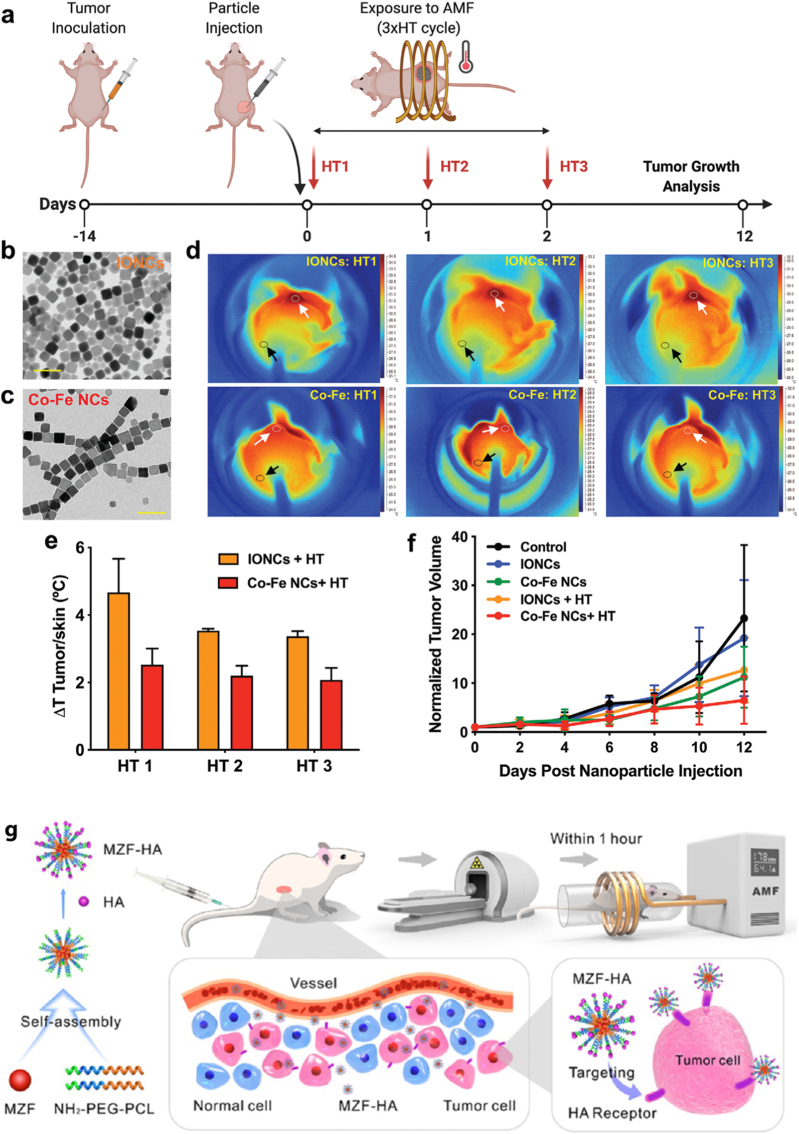
(a) The schematic outlines the treatment process, where the black arrow indicates the injection of nanoparticles (0.7 mg of Co–Fe NCs or IONCs), and the red arrows show three hyperthermia (HT) sessions under clinically approved AMF conditions (frequency: 110 kHz; field strength: 20 kAm^−1^). (b, c) TEM images display the morphology of the nanoparticles used in the study, with IONCs averaging 18 ± 3 nm and PMAO-coated Co–Fe NCs measuring 17 ± 2 nm (scale bar: 50 nm). (d) Infrared images captured during HT on days 1, 2, and 3 (HT1, HT2, HT3) highlight temperature differences between the tumor (white arrow) and skin (black arrow). (e) The ΔT graph (ΔT = T_Tumor_ − T_Skin_) compares the temperature increase for each HT session, with orange bars representing IONCs and red bars representing Co–Fe NCs. (f) The tumor growth curve indicates a more substantial reduction in tumor size for mice treated with Co–Fe NCs combined with HT compared to other groups, with a sample size of six (N = 6). Reprinted with permission from Ref. [161], Copyright 2020 John Wiley and Sons [161]. (g) Diagram showing Mn-Zn ferrite magnetic nanoparticles functionalized with hyaluronic acid for cancer therapy. Reproduced with permission from Ref. [162]. Copyright 2020, American Chemical Society. (For interpretation of the references to color in this figure legend, the reader is referred to the Web version of this article.)

**Magnetic fields responsive systems:** Magnetic field-responsive nanostructures have garnered significant attention for their ability to provide precise control over drug release in response to external magnetic stimuli. These systems typically employ magnetic nanoparticles (MNPs) [2], such as iron oxide nanoparticles [155], which can be functionalized with polymers or biomolecules to achieve enhanced stability, biocompatibility, and targeted delivery. Upon exposure to an external magnetic field, MNPs can generate localized heat (via magnetic hyperthermia) or undergo mechanical motion, facilitating the disruption of carrier matrices and the subsequent release of therapeutic agents [156]. Furthermore, alternating magnetic fields can be utilized to induce on-demand, remote-controlled drug delivery, minimizing systemic side effects and enhancing therapeutic efficacy. Recent advancements have integrated these systems with imaging modalities, such as magnetic resonance imaging (MRI), enabling real-time tracking and theranostic applications [157]. The ability of magnetic field-responsive nanostructures to combine spatiotemporal control with multifunctionality makes them promising candidates for next-generation controlled release systems in oncology, regenerative medicine, and beyond [158]. Li et al. [159] developed PSiNPs@(Fe_3_O_4_/Au) nanocomposites by combining iron oxide/gold nanoparticles with porous silicon nanoparticles. These nanocomposites showed superparamagnetism, photothermal effects, and magnetic resonance imaging capabilities [159]. They enhanced drug delivery to drug-resistant breast cancer cells with a magnetic field and NIR light, improving cellular uptake, drug release, and therapeutic efficacy [159]. Cho et al. [160] developed Janus microcarriers for targeted combination chemotherapy of hepatocellular carcinoma. These carriers, containing regorafenib and doxorubicin in separate compartments, use magnetic fields for controlled drug release and offer MRI capabilities for precise tumor delivery [160]. Balakrishnan et al. [161] evaluated the therapeutic potential of cobalt ferrite nanoparticles (Co–Fe NCs) compared to iron oxide nanoparticles (IONCs) in combination with magnetic hyperthermia (HT) for cancer treatment. Fig. 3a illustrates the experimental timeline, where tumor-bearing mice received nanoparticle injections (0.7 mg of IONCs or Co–Fe NCs) followed by three cycles of HT under clinically approved alternating magnetic field (AMF) conditions (frequency = 110 kHz; field strength = 20 kA m^−1^). Fig. 3b and c displays transmission electron microscopy (TEM) images showing the cubic morphology of Co–Fe NCs (17 ± 2 nm) and the spherical structure of IONCs (18 ± 3 nm) [161]. Infrared thermal imaging in Fig. 3d reveals a higher localized temperature at tumor sites for Co–Fe NC-treated mice across all three HT sessions (HT1, HT2, HT3), as indicated by the black and white arrows marking tumor and skin temperatures, respectively. The temperature differential (ΔT = Ttumor – Tskin) is quantified in Fig. 3e, where Co–Fe NCs generates a more substantial thermal increase compared to IONCs. This enhanced heat generation, combined with the inherent cobalt toxicity and unique chain formation of Co–Fe NCs under AMF, leads to a significant reduction in tumor volume. As shown in Fig. 3f, the tumor growth curve demonstrates that the Co–Fe NCs + HT group exhibits the most pronounced tumor shrinkage, highlighting the superior therapeutic efficacy of this dual-action platform [161]. Wang et al. [162] developed a nanoparticle-based system to enhance radiotherapy (RT) for non-small-cell lung cancer (NSCLC) by combining it with hyperthermia (HT). They used Mn–Zn ferrite magnetic nanoparticles (MZF) loaded in PEG-PCL micelles modified with hyaluronic acid (HA) for targeted delivery to CD44-overexpressing A549 tumor cells. Under an alternating magnetic field (AMF), MZF generated localized heat (∼43 °C), improving tumor oxygenation and enhancing RT effects. In vitro and in vivo studies demonstrated effective tumor targeting, increased apoptosis, and significant tumor volume reduction (49.6%) with combined HT and RT, suggesting the potential of MZF-HA for improved NSCLC treatment (Fig. 3g) [162]. Alongside material innovations, AI is increasingly contributing to the rational engineering and optimization of external stimuli-responsive nanocarriers [124]. In temperature-responsive systems, ML models have been applied to predict LCST behavior, phase transition thresholds, and drug release kinetics based on polymer composition, molecular weight, and grafting density [124]. For light-responsive platforms, DL approaches assist in optimizing photosensitizer loading, photothermal conversion efficiency, and irradiation parameters to balance therapeutic efficacy and tissue safety [163]. Data-driven modeling can further support the design of photocleavable linkers and up-conversion nanoparticle architectures by correlating structural descriptors with light-triggered release performance. In magnetic field-responsive systems, surrogate modeling and Bayesian optimization frameworks enable prediction of heat generation efficiency, magnetic hyperthermia thresholds, and field-dependent release dynamics under alternating magnetic field conditions. Importantly, AI-assisted simulation can integrate material properties with physical stimulus parameters (e.g., wavelength, field strength, exposure duration) to achieve spatiotemporally controlled drug release while minimizing off-target damage [124,163]. Although comprehensive AI methodologies are discussed in section 3, their integration within external stimuli-responsive systems reflects a shift toward predictive, parameter-aware design strategies that couple material engineering with stimulus modulation. AI algorithms could refine designs of magnetic nanoparticle carriers (such as Fe_3_O_4_, ferrite). Input parameters could comprise the size of magnetic cores, coating thicknesses, strengths and frequencies of external fields, and viscosities of suspensions; output variables include estimated rates of temperature increase (SAR), torque generated by the magnetic field, and/or dissociation induced by an external field. SAR prediction models for superparamagnetic particles can be trained using ANNs to select optimal particle geometry for inducing hyperthermia. While direct applications of AI techniques are limited, the capability of ML algorithms to correlate NP properties with their effectiveness implies that AI could adjust magnetic sensitivity [46,164,165].

Thus, the role of AI in every stimulus is that it possesses the ability to handle the complexities and make predictions regarding the outcome. This is done through the use of data vectors where the AI (neural networks/tree ensembles/generative models) can be used to come up with some insights or optimal formulation for the stimuli. Table 3 is a comparison of key aspects of the stimuli.

**Table 3:** Summary of AI input features, output targets, and functional roles in stimuli-responsive drug delivery systems.

Ref	AI Role (beyond release)	Output Targets	Typical Input Features	Stimulus
[45,46]	Designing acid-sensitive polymers or micelles; optimizing pH threshold and release kinetics	Optimized polymer formulation, target pKa/LCST, release onset pH, loading efficiency	Polymer composition (monomer ratios, pKa, MW), block lengths, zeta-potential, environmental pH conditions	pH-responsive
[46,59,122]	Designing redox-labile linkers and composite carriers; tuning release threshold and rate under reducing conditions	Degradation/cleavage rate, GSH-triggered release profile, stability under oxidizing conditions	Redox-labile bond density (disulfide/thioketal content), polymer hydrophobicity, reductant (GSH/ROS) concentration	Redox-responsive
[121,122]	Designing enzyme-cleavable sequences; optimizing substrate specificity and loading sites for on-demand release	Cleavage fraction, release onset time, carrier morphology change	Peptide linker sequence (cleavage motif), enzyme concentration/activity, polymer matrix properties	Enzyme-responsive
[46,59]	Tuning polymer LCST (e.g. via copolymerization); optimizing particle/gel structure for sharp thermal response and controlled phase-change release	Transition temperature (LCST/UCST), swelling ratio, release burst above threshold, photothermal conversion efficiency	Polymer composition (monomers, MW, crystallinity), LCST/UCST values, crosslinking density, thermal conductivity, ambient temperature profile	Temperature-responsive
[46,58]	Designing photosensitive molecules or photothermal agents; optimizing irradiation parameters for pulse/remote release; integrating light and chemo effects	Photo-triggered bond cleavage or conformational change, generated heat or ROS, release timing/dose, photothermal temperature	Chromophore/NP optical properties (absorption λ, quantum yield), particle composition (e.g. MoS_2_, Au), light wavelength/intensity, irradiation time	Light-responsive
[46,59,122]	Engineering magnetic nanocarrier geometry for efficient hyperthermia or mechanical actuation; optimizing field parameters for on-demand release (e.g. hyperthermia-assisted release)	Specific absorption rate (SAR, heating rate), particle rotation/force, induced drug diffusion, alignment	Magnetic core composition (Fe_3_O_4_, ferrite), particle size/shape, coating thickness, field strength/frequency, medium viscosity	Magnetic-responsive

## 3 Integration of AI in stimuli-responsive drug delivery systems

The formulation of nanocarriers for targeted drug delivery requires a holistic understanding of material composition, interfacial physicochemical properties, and dynamic biological interactions. Traditionally, formulation approaches have been largely dependent on trial-and-error experimentation, which is time-consuming and, more often than not, lacks predictive capability. The integration of AI, and more specifically, ML, brings with it a data-driven paradigm that has the ability to identify complex, non-linear correlations within high-dimensional data. By harnessing the power of these computational resources, researchers can now make direct connections between material properties and functional performance [163]. This paradigm embodies the rational principles of nanoarchitectonics, whereby functional nanostructures are designed and assembled using bottom-up approaches instead of empirical optimization. In this regard, AI serves as an enabler of high-throughput screening, formulation optimization, and performance prediction [166,167]. The general workflow of AI-assisted nanocarrier design includes the following steps: The first step involves the creation of a set of formulation candidates based on material libraries with different characteristics [168]. Such properties affect important therapeutic factors such as the loading efficiency of drugs, release kinetics in response to certain stimuli, targeting efficacy, and biocompatibility. The results of experiments conducted using these libraries are used to train ML algorithms, which help to discern complex correlations between the variables of drug formulation and delivery performance. After validation, the trained models help to make informed modifications to the nanocarrier structure to meet certain therapeutic needs. AI thus helps to make a shift from empirical formulation to mechanism-based and possibly personalized drug delivery [163].

### 3.1 AI in material and carrier design

The design of stimuli-responsive nanocarriers is a complex multivariable optimization problem, with nonlinear structure-response relationships, crosstalk between stimuli, and limited experimental data, making rational formulation development difficult. Conventional trial-and-error methods are inefficient and time-consuming for exploring the high-dimensional design space. In this scenario, AI offers data-driven solutions for predictive modeling, inverse design, and automated optimization, thus enabling the development of smart drug delivery systems from empirical screening to computationally assisted and closed-loop approaches [169,170]. AI-driven models (including ML and DL) are increasingly applied to the design of stimuli-responsive nanocarriers. These models can mine high-dimensional compositional data to establish structure–property relationships, enabling *in silico* prediction of carrier behavior under external (such as temperature, light) or internal (pH, enzymes) triggers. For example, ML-enabled simulations can predict how a hydrogel will swell, degrade, or release payload in response to pH or temperature changes [171]. AI algorithms also rapidly screen polymer and cross-linker combinations to achieve targeted mechanical or chemical properties, tailoring carriers for specific stimuli [172,173]. By guiding high-throughput formulation searches, AI accelerates the discovery of materials with desired responsiveness (such as new pH- or redox-responsive gels) from vast candidate libraries [173].

Supervised learning has been used to predict nanocarrier properties directly from formulation descriptors. For instance, ML models trained on poly(2-oxazoline) micelle data accurately predicted drug-loading efficiency (balanced accuracy ∼0.8) across different polymer compositions [174]. Similarly, an ensemble-tree ML framework trained on thousands of lipid nanoparticle (LNP) formulations achieved >90% accuracy in classifying gene-delivery activity and cell viability [175]. Feature-attribution analyses, including SHAP, identified the key physicochemical parameters governing LNP performance, such as particle size, surface charge, and lipid composition. These insights enabled more rational and data-driven formulation design [175]. In parallel, genetic algorithms combined with ML surrogate models have been used to address inverse design challenges. Rather than predicting properties from existing materials, these approaches evolve chemical structures to meet predefined target properties. For example, polymer chemistries were optimized to achieve simultaneously high glass-transition temperatures and large bandgaps, leading to the discovery of novel materials and actionable chemical design rules [176]. Collectively, these studies demonstrate how supervised AI methods can efficiently navigate complex, multivariate design spaces, predict material performance, and accelerate the identification of optimal formulations.

Unsupervised learning and optimization strategies further complement supervised approaches by identifying latent material patterns and efficiently navigating complex formulation spaces. Dimensionality reduction techniques, such as principal component analysis (PCA), together with clustering algorithms, can reveal hidden groupings of polymers or hydrogels based on their chemical and functional features [177]. These analyses facilitate feature selection and support the rational design of material libraries. In addition, active-learning strategies—particularly Bayesian optimization—enable iterative refinement of synthesis parameters to achieve predefined target behaviors. For example, a Bayesian–deep neural network framework was applied to optimize nanoparticle synthesis conditions in a microfluidic platform, ensuring that the resulting particles matched a desired absorbance spectrum [178]. In another study, molecular dynamics simulations of drug–excipient interactions were integrated with ML and high-throughput screening to identify drug–excipient combinations capable of forming highly stable nanoparticles [178].

These AI-assisted strategies substantially reduce the experimental search space by prioritizing the most promising formulations for validation. By shifting from empirical trial-and-error approaches to predictive modeling and rational design, researchers can evaluate untested formulations computationally before physical experimentation [171]. This significantly decreases the number of required laboratory experiments and accelerates material discovery. Novel polymer chemistries and nanocarrier compositions can therefore be screened *in silico* for stimuli-responsiveness prior to bench-scale testing. Importantly, many AI models also provide interpretable outputs, uncovering structure–function relationships that inform the a priori design of carriers with defined stimuli sensitivity [175].

In nanocarrier and material design, various AI models have their own strengths and weaknesses. Deep convolutional neural networks (CNNs) have the ability to learn complex spatial or structural patterns (e.g., from microscopy images or molecular structure graphs), but they need large amounts of high-quality data and are often considered “black box” models [163,179]. Likewise, generative models (generative adversarial networks (GANs), variational autoencoders (VAEs)) facilitate reverse design by suggesting new nanocarrier designs with specific properties, but they are highly data-intensive and tend to generate a large number of invalid designs that need to be removed by experts [163]. In contrast, tree-based ensemble algorithms (Random Forest, gradient boosting) are very efficient in training on a medium-sized tabular data and provide inherent robustness and interpretability. These have been successful in the formulation tasks: for instance, LightGBM/XGBoost was able to predict the release of drugs from a polymeric long-acting injectable formulation with R^2 ≈ 0.92 [169], and LSBoot was able to accurately model the liposomal particle size and polydispersity for different preparation conditions [180]. Tree-based methods are likely to be superior to linear models in nonlinear formulation spaces by bagging or boosting higher-order terms [169], although they might have difficulty leveraging direct structural inputs unless large amounts of data are available. Lastly, Bayesian optimization, coupled with an ML proxy, is highly effective at informing experimental design by effectively navigating high-dimensional formulation or hyperparameter spaces. Bayesian optimization has been employed in closed-loop experimental series to optimize polymer-based nanocarriers, such as in a recent study that utilized Bayesian optimization to filter through hundreds of polymer formulations to find the best ones for nucleic acid delivery [181].

In reality, the choice of algorithm depends on the type of data modality and the objective. CNNs (or a combination of CNNs and LSTMs) are best suited for image-dense or sequence data (for example, analyzing nanocarrier morphologies or time-series stability profiles) [179]. Generative models are more suited to creative, exploratory design of new materials, but at the expense of needing a lot of data and validation [182]. Tree-ensembles and Gaussian processes excel when data are limited: they enable precise predictions of properties or performance from standard formulation descriptions and easily assess the importance of features [179]. Bayesian methods, on the other hand, do not make predictions of performance but efficiently suggest the next experiments or candidates, trading off multiple objectives (such as potency versus stability). In polymeric and stimuli-responsive nanomaterials, where design spaces are complex but data are limited, new hybrid strategies are being developed: for instance, combining interpretable ensembles with Bayesian loops or reinforcement learning (RL) to iteratively optimize smart DDS formulations [169]. Overall, no individual AI strategy is superior to the rest: CNNs and DL need big data to outperform simpler models [179], while tree ensembles and Bayesian optimization offer solid, interpretable starting points for most nanocarrier design tasks [169,181]. In conclusion, ML and DL tools are transforming carrier design in nanomedicine by enabling rapid screening, predictive formulation optimization, and rational engineering of smart drug-delivery materials.

### 3.2 AI in predictive modeling and simulation

ML and DL are increasingly applied to simulate and predict the dynamic behavior of stimuli-responsive nanocarriers. Data-driven models can capture complex, non‐linear relationships between carrier composition, environmental triggers (pH, redox, temperature) and drug release kinetics, supplementing traditional physics-based simulations [183,184]. For example, Gao et al. [183] note that ML can extend the efficiency and predictive power of molecular simulations for polymeric drug carriers under various stimuli [183]. In practice, AI-augmented modeling allows high‐throughput virtual screening of formulations and conditions that would be infeasible with experiments alone.

Surrogate modeling of nanoscale simulations has shown dramatic speedups. In one study, a DL framework was trained to forecast the solvent-accessible surface area (SASA) of drug-loaded nanoparticles hundreds of timesteps into the future, achieving ∼40–300 × faster predictions than full molecular dynamics (MD) runs while improving accuracy [184]. This kind of ML-MD integration compresses costly atomic‐level simulations into efficient predictive pipelines. Similarly, trained ML regressors can predict drug diffusion or release rates in response to environmental changes; for instance, tree-based and neural models accurately reproduced measured release profiles of long-acting polymeric injectables, effectively guiding formulation design without exhaustive lab tests [185]. These cases illustrate that AI models can learn stimulus–response kinetics directly from data, forecasting entire release curves under new pH or temperature conditions.

Various ML architectures have been employed to model nanocarrier–stimulus interactions. Feedforward and CNNs, recurrent neural networks (RNN)/long short-term memory (LSTM) models for time series, random forests, SVMs, Gaussian processes and even graph neural networks have been used to map input features (polymer type, carrier size, stimulus level) to outputs (release rate, swelling ratio, stability) [171,184,186]. For example, Negut et al. [171] review numerous ML techniques—ANN, SVM, RF, CNN, GAN and RL—applied to stimuli-responsive hydrogels and nanogels. In their survey, CNNs achieved >90% accuracy in modeling hydrogel permeability, while GANs/RL accelerated the formulation of self-healing, stimuli-triggered polymer networks [171]. These studies demonstrate that advanced AI methods can both predict complex release behavior and even generate novel nanocarrier designs with tailored stimulus-sensitivity.

Looking forward, combining ML/DL with multiscale physics models and experimental feedback loops promises further gains. AI-driven “digital twin” models could integrate patient-specific physiological data and nano–bio interaction models to personalize carrier design and dosing schedules. Challenges remain in data scarcity and model interpretability; thus, future work will emphasize explainable AI (XAI) and physics-informed ML to ensure reliable predictions. Overall, AI-enabled simulation frameworks are rapidly improving our ability to understand and control nanocarrier dynamics under pH, redox, thermal and other triggers, paving the way for more precise and efficient smart drug delivery systems [171,184].

More recent modeling strategies are further augmenting predictive nanocarrier simulations. For example, physics‐informed neural networks (PINNs) embed known mechanistic equations (diffusion, transport) into the learning process, allowing models to simulate nanoparticle transport or drug release while respecting physical laws [187]. Transfer learning leverages knowledge from larger, related datasets by fine‐tuning a pretrained model on a new, smaller nanocarrier dataset, thereby reducing data requirements and accelerating training [188]. Finally, uncertainty quantification (UQ) methods (Gaussian process regression or Bayesian neural nets) provide predictive confidence bounds. For instance, GPR has been used to predict nanoparticle properties while naturally yielding a measure of predictive variance, which is crucial when data are scarce [189]. By integrating PINNs, transfer learning, and UQ, next‐generation AI models can produce more reliable, interpretable, and robust simulations of stimuli-responsive nanocarriers.

### 3.3 AI for multi-stimuli optimization

Stimuli-responsive nanocarriers that react to two or more triggers (such as pH, temperature, redox, enzyme) offer powerful on-demand drug release, but their design is challenging. Multi-stimuli systems require combining distinct responsive chemistries, which greatly increases formulation complexity and often compromises stability [190]. Ensuring that each stimulus produces a synergistic and predictable release (rather than triggering premature or competing release) is difficult. Multi-functional carriers can also raise safety concerns (new labile bonds or particles) and manufacturing reproducibility issues [190]. Moreover, tuning a multi-stimulus platform involves many variables (monomer types/ratios, copolymer architecture, particle size, surface chemistry, etc.), leading to a combinatorial large optimization space that is impractical to search by trial and error.

Traditional formulation development thus struggles with high-dimensional, multi-objective design problems in this context. ML and AI can address these challenges by learning structure–property relationships and guiding search in large parameter spaces. In silico ML models can map carrier composition and structure to functional outcomes, enabling rapid screening and optimization. For example, Hayakawa et al. [191] built a sparse regression model to predict the cloud-point temperature of thermo-responsive copolymers, effectively guiding the design of new polymers with desired thermal trigger points [191]. Similarly, Kehrein et al. [192]compiled data on poly(2-oxazoline)/poly(2-oxazine) micelles and trained a range of ML regressors to predict drug loading. The best models achieved balanced accuracies ≈0.8 and revealed which polymer and drug features most influence loading [192]. These examples show that ML can discover nonlinear relationships in large formulation datasets. Advanced techniques like Bayesian optimization can further accelerate design: for instance, Gormley and Webb used Bayesian ML to select monomer ratios for copolymers, yielding polymer–protein hybrids with enhanced thermal stability [193]. In general, AI supports multi-objective optimization (such as balancing release rate vs. loading vs. stability) by learning Pareto‐optimal trade-offs across many parameters.

Experimentally, AI/ML has begun to integrate with formulation workflows for multi-stimuli carriers. Yanes et al. [194] compiled an extensive database of liposome formulations and in vitro test conditions, then developed an ML workflow linking formulation attributes (lipid composition, size, etc.) and IV release test parameters (pH, temperature, stirring) to drug release profiles [194]. Their classification model could predict the kinetic release class from these inputs, thus informing the design of liposome release studies. In another study, Sun et al. [195] combined ML with bench experiments on PLGA nanoparticles: they first analyzed literature data (∼50 studies) to see how drug solubility, molecular weight, particle size, and medium pH affect release. The ML-driven insights were then used to plan new in vitro release experiments, which indeed confirmed the model's predictions (such as acidic pH accelerating release) [195]. In a third example, AlOmari et al. [196] collected Raman spectroscopy data from polysaccharide-coated tablets and used ML regressors (elastic net and neural nets) to predict colon-targeted release of 5-ASA. The multilayer perceptron model achieved R^2^ ~ 0.999 on the test set [196], showing that AI can accurately predict multi-condition (stomach vs. intestinal pH) release behavior from high-dimensional input data. These studies exemplify how ML can assist multi-stimuli formulation by relating many formulation/test variables to release outcomes, and then optimizing formulations before or during experimental validation.

AI and ML algorithms are very efficient in learning complex nonlinear relationships in high-dimensional design spaces [163,197]. In the context of multi-stimuli-responsive carriers, this enables “multivariate” models to learn about the high-order interactions between the stimuli and material properties (such as pH, redox, enzyme responsiveness, and so on). For instance, nanoarchitecture design enables the design of logic-gated release systems that respond only when particular combinations or sequences of stimuli are available [20,21]. Then, ML can be employed to forecast whether particular stimuli will work in synergy or interfere with each other – essentially distinguishing between orthogonal (non-overlapping) trigger combinations and those that could potentially cause crosstalk. In other words, AI-based methods have been demonstrated to speed up carrier design and optimize the release kinetics in complex multi-input systems [197,198], making it possible to rationally design orthogonal or hierarchically gated release mechanisms. In the case of cascade or staged release designs, in which one trigger “primes” the system for a later activation, time-dependent models are clearly critical. Recurrent neural networks and other dynamic ML models can be trained on kinetic data to model how a nanocarrier might respond to a series of stimuli over time. By incorporating release profiles into a predictive model, AI models can predict the temporal release profile under physiologically relevant conditions. This enables scientists to design staged liberation of drugs (for example, first an anti-inflammatory, then a chemotherapeutic) without necessarily resorting to trial-and-error. In general, AI/ML models have been demonstrated to greatly accelerate and improve nanocarrier design by extracting time-function relationships from data [163,199,200]. Lastly, incorporating multiple modules that respond to stimuli is bound to introduce trade-offs (for example, the presence of more labile bonds may lower the stability of the carrier). AI can optimize these conflicting requirements by formulating the design problem as a multi-objective optimization problem. For example, Bayesian optimization and Pareto front analysis have been used in nanoparticle design to find a formulation that strikes the best possible balance between drug loading, sensitivity to the trigger, and toxicity [199,201]. For instance, in a study, multi-objective Bayesian optimization identified a Pareto optimal design region for curcumin nanocarriers, obtaining ∼82% reduced toxicity with ∼70% retained drug loading [201]. In reality, these ML-assisted pipelines enable scientists to quantitatively explore the stability-complexity plane: they can estimate the impact of incorporating a new responsive linker or targeting ligand on trigger responsiveness but also on circulation half-life or immunogenicity. By sorting potential carriers on a Pareto front of, for example, sensitivity and stability, AI assists in choosing an optimal trade-off that would be very hard to identify through brute-force experimentation [200,201]. Taken together, these AI-informed approaches turn the design of multi-stimuli nanocarriers from a computationally intractable problem of combinatorial optimization into a data-driven process. By learning from large multidimensional datasets (or high-throughput simulations) and making predictions about complex behaviors (crosstalk, cascade release times, stability), AI offers a systems-level approach that enables the rational integration of multiple stimuli without compromising stability [20,202]. In conclusion, AI provides powerful tools to tackle the complexity of multi-stimuli nanocarrier design. Data-driven models and optimization algorithms can search vast combinatorial spaces and balance multiple objectives, effectively “closing the loop” between design and performance. By learning structure–property mappings, AI can suggest polymer sequences or nanoparticle compositions that yield desired dual-trigger responses. For instance, one could train a model on thermo/pH‐responsive polymer blocks and use it to predict formulations that respond only when both triggers are present. In practice, AI-enabled frameworks are beginning to tame the complexity of these systems. Recent literature even demonstrates sophisticated multi-responsive DDS (such as thermo/pH/magnetic hydrogels and magneto-responsive liposomes) [190,203]. AI-driven workflows aim to systematically optimize such platforms. In essence, AI and ML allow multi-stimuli carrier design to be framed as high-dimensional, multi-objective optimization problems – problems that can be solved more efficiently by learning from data than by intuition alone [191]. This intelligent approach accelerates the development of tailored, responsive nanocarriers.

### 3.4 AI in data integration and personalized therapy

Recent advances in AI have enabled the fusion of diverse biomedical datasets to tailor drug delivery to individual patients. For example, Simon et al. [134] demonstrated that multimodal AI models can simultaneously analyze imaging data and electronic health records to guide cancer therapy planning [134]. By combining genomic, transcriptomic, proteomic, and clinical features in a unified model, AI can identify patient-specific biomarkers and treatment targets. In one study Samathoti et al. [204] describe how AI algorithms analyze genomic, proteomic and clinical data to formulate personalized nanoparticle-based therapies. These integrative models predict how individual tumors will respond to different formulations, enabling selection of the optimal nanocarrier composition (size, surface ligands, drug cargo, release kinetics) for each patient. This multimodal data integration directly informs the design and optimization of stimuli-responsive nanocarriers. For instance, AI can identify tumor-specific triggers (such as pH, enzyme overexpression, or receptor profiles) from genomics and imaging data, and then optimize carrier properties accordingly. AI-powered *in silico* models can simulate nanoparticle behavior (biodistribution, tumor accumulation, clearance) in virtual patient scenarios. Samathoti et al. [204] report that predictive models can forecast NP interactions in vivo – for example, adjusting surface chemistry to improve tumor uptake and reduce off-target effects [204]. An illustrative case is the AI-driven design of a mesoporous silica nanoparticle loaded with cisplatin: by training on preclinical datasets, the AI fine-tuned pore size and surface groups to achieve targeted drug release. In hepatocellular carcinoma models, these AI-optimized MSNs achieved ∼60% greater tumor reduction than conventional formulations [205]. Similarly, AI has optimized lipid nanoparticle formulations: one example involved designing LNPs to preferentially target KRAS-mutant tumor cells. By analyzing mutation-specific signaling pathways and patient tumor profiles, the AI selected novel lipid combinations and ligands, resulting in enhanced specificity for KRAS-driven cancers and reduced systemic toxicity [205]. These examples illustrate how linked “big data” – including genomics, proteomics, imaging, and even longitudinal sensor (wearable) data – feed into carrier design. AI identifies the most predictive molecular and microenvironmental features, and proposes nanocarrier architectures that release drugs in response to those patient-specific cues (such as enzyme-cleavable bonds in the tumor microenvironment or receptor-targeted delivery) [204,205].

Methodologically, the AI/ML toolkit for personalized nanomedicine includes advanced strategies for learning from limited, heterogeneous data.

In a recent advance, Shan et al. [206] proposed a data-driven approach to directed evolution that integrates the design of virtual nanoparticles, combinatorial synthesis, (Deoxyribonucleic acid) DNA/peptide barcoding-assisted in vivo screening, and ML-based structure-activity modeling. This approach replaces traditional one-dimensional screening with an adaptive optimization loop, in which biological performance data are used to iteratively inform design cycles. By integrating high-throughput experimentation with computational learning, this approach systematically unravels nano-bio interaction patterns and optimizes nanoparticle architecture for improved delivery efficiency. These integrated experimental-computational models of evolution are a crucial step towards autonomous AI-assisted nanomedicine discovery [206]. Xue et al. [207] developed an AI-Guided Ionizable Lipid Engineering (AGILE) platform to fast-track the discovery of lipid nanoparticles (LNPs) for mRNA delivery. The platform combines the capabilities of combinatorial lipid synthesis with a pre-trained graph neural network (GNN) model that was trained on a large dataset of thousands of measurements of LNP activity [181]. By computationally extrapolating from the large number of experimentally validated libraries, the model was able to screen and rank the expanded chemical space well beyond the initial set of synthesized lipids, thus allowing the discovery of formulations with improved transfection efficiency. This approach represents a hybrid method of how ML-assisted exploration of structure-function relationships can greatly improve the optimization of LNPs and help to develop extrahepatic mRNA delivery systems, such as lung-targeted platforms [207]. Recent breakthroughs also demonstrate the revolutionary potential of DL in the design of ionizable lipids. Witten et al. [208] developed a lipid optimization tool based on a neural network, trained on a set of over 9000 activity measurements of lipid nanoparticle (LNP) compounds. By using a directed message-passing neural network, they were able to perform large-scale *in silico* screening of approximately 1.6 million candidate lipids, successfully identifying new molecules with the ability to deliver mRNA efficiently to muscle, nasal mucosa, and lung tissues in vivo. To complement this strategy, Xu et al. [209] created the AI-Guided Ionizable Lipid Engineering (AGILE) platform, which combines deep neural networks with combinatorial lipid synthesis to facilitate the rapid discovery of cell-specific LNP formulations. Their strategy showed that computational models can reveal cell type-specific structure-function preferences, allowing for the rapid design and selection of lipids optimized for various biological targets. These studies together illustrate how the use of AI-assisted exploration of large chemical spaces, in combination with high-dimensional structure-activity modeling, can greatly facilitate the design of tailored, application-specific mRNA delivery systems [208,209].

Transfer learning is widely applied: Hao et al. [210] note that fine-tuning neural networks pretrained on large image or biological datasets can dramatically speed convergence on new, small biomedical datasets [210,211]. In practice, a model pretrained on a large drug or nanoparticle database can be adapted to a specific patient's data with few additional examples. Active learning is also employed to efficiently explore the vast design space of nanocarriers: the AI model iteratively selects the most informative experiments to perform next, thus learning optimal formulations with minimal experimental effort [210,212]. For example, in nanoparticle formulation optimization, active learning can choose which combinations of ingredients to test in the next lab batch to improve the model of drug release or targeting. Uncertainty quantification techniques (for example bayesian neural networks, ensemble methods, or Monte Carlo dropout) are incorporated to assess the confidence of AI predictions. Quantifying uncertainty is crucial when planning therapy: it helps flag cases where the model's recommendation may be unreliable (due to sparse data or out-of-distribution inputs) and guides further data collection. By explicitly modeling uncertainty, AI systems can communicate confidence levels in predicted biodistribution or efficacy, which supports risk-aware decision making [188,212]. Despite these advances, significant challenges remain. Data heterogeneity is a major obstacle: genomic, proteomic, imaging, clinical record and sensor data all have different formats, scales, and noise characteristics. Integrating such diverse modalities often requires careful normalization and data curation. Missing or biased data in any modality can skew the AI models. For example, wearable sensor streams may be interrupted, and batch effects in sequencing can confound associations. Overcoming these issues requires sophisticated data harmonization and robust modeling techniques. Another key challenge is model interpretability as noted by Serrano et al. [213], many AI models remain “black boxes,” making it difficult to trace why a particular carrier design or therapy plan is recommended [213]. Clinicians and regulators demand transparent decision logic, especially for personalized treatments. XAI methods are being explored to highlight which biomarkers or patient features drive the AI's choices, but this remains an active area of research. In summary, AI-driven data integration holds great promise for tailoring stimuli-responsive drug delivery systems to individual patients. Continued methodological innovation (for example improved transfer learning and active learning schemes) and emphasis on interpretability and robust uncertainty estimation will be essential to translate these AI models into reliable clinical practice [213].

However, *in silico*, AI-assisted designs can be challenging in real-world personalized medicine. The DL models are vulnerable to overfitting and may learn spurious relationships from incompletely specified or biased training populations [214,215]. Patient datasets coming from different sources are heterogeneous in terms of data type, size, and quality (genomic, proteomic, imaging, and EHR), and this leads to distributional (domain) shifts from training to testing, causing a loss of model performance [215,216]. Technical biases, such as sequencing batch effects [191] or imbalanced patient populations, can introduce additional spurious relationships that are not revealed by standard validation procedures [216]. In reality, many models are still “black boxes” developed on small, single-center datasets, so without proper external validation, they can silently fail on new populations [215]. In fact, performance measures on small test sets can be misleadingly high if the test set shares hidden biases with the training set [216]. This has led to very few AI-based nanocarrier systems being prospectively tested or generally accepted in the clinic, with only a small number of models being able to translate into real-world applications [217]. This lack of evidence creates a problem for the translation of regulatory frameworks, as there is a lack of well-developed frameworks and a problem of liability in proving the safety and efficacy of AI systems that are constantly learning [218].

New methodological developments are emerging to address these challenges. Federated learning allows for decentralized multi-center training on private patient data, which can enhance robustness to heterogeneous populations while maintaining privacy. [216,217]. Methods for uncertainty quantification (e.g., Bayesian or ensemble learning) enable principled quantification of confidence, indicating regions of low confidence or out-of-distribution predictions and informing data collection [219]. Reporting guidelines (TRIPOD-AI for predictive models, CONSORT-AI for AI trials) are being adopted to ensure compliance with transparency, completeness, and reproducibility [220,221]. In fact, structured implementation frameworks (such as SALIENT) have been proposed to incorporate reporting guidelines into clinical pipelines (from retrospective validation to RCT) [218]. For instance, CONSORT-AI specifically states the need for transparent reporting of AI-related trial design features to enable critical evaluation [221], while TRIPOD-AI improves the conventional reporting of prognostic models to incorporate ML information [220]. Taken together, these measures, in addition to external validation, domain adaptation, and XAI software, hold the promise of enhancing the validity, safety, and credibility of AI-assisted personalized nanocarrier therapies [219,222]. Such a multifaceted approach is critical to meet the regulatory requirements and gain the confidence of clinicians and patients in AI-assisted therapies. To offer a systematic insight into the AI techniques currently used in the design of drug delivery systems, Table 4 lists the major computational techniques, their implementation approaches, associated tasks, and inherent strengths and weaknesses. These technologies include predictive modeling, generative models, optimization algorithms, and interpretability algorithms, which are used for the optimization of nanoparticle formulation, structure-property relationships, and performance prediction. By analyzing the algorithmic differences and implementation limitations, the table allows for a critical assessment of their suitability based on the nature and availability of data. Compared to the traditional trial-and-error approach, which involves extensive experimental screening, the AI-assisted approach allows for predictive modeling, optimization, and rational material selection, thus accelerating the formulation development process.

**Table 4:** Overview of current AI technologies in drug delivery system design: applications, strengths, and limitations.

Ref	Limitations	Advantages	Representative Tasks	Role of AI in Drug Delivery	AI Technology
[189,192]	Limited with high-dimensional data	Interpretable, works with small datasets	Property prediction, screening	Predict nanoparticle size, zeta potential, drug loading	ML
[223,224]	Data-hungry, low interpretability	Captures complex nonlinearities	Imaging analysis, biodistribution prediction	Model nonlinear synthesis–performance relationships	DL
[225,226]	Training instability	Enables de novo design	Chemical space exploration	Generate novel nanocarrier compositions	GANs
[227,228]	May oversimplify structure	Smooth latent representation	Structure optimization	Latent space exploration of nanostructures	VAEs
[229,230]	Computationally intensive	Dynamic optimization	Sequential synthesis optimization	Optimize formulation iteratively	RL
[231,232]	Adds computational complexity	Improves trust	Regulatory transparency	Interpret black-box models	XAI

## 4 Biomedical application

**Fig. 4:**
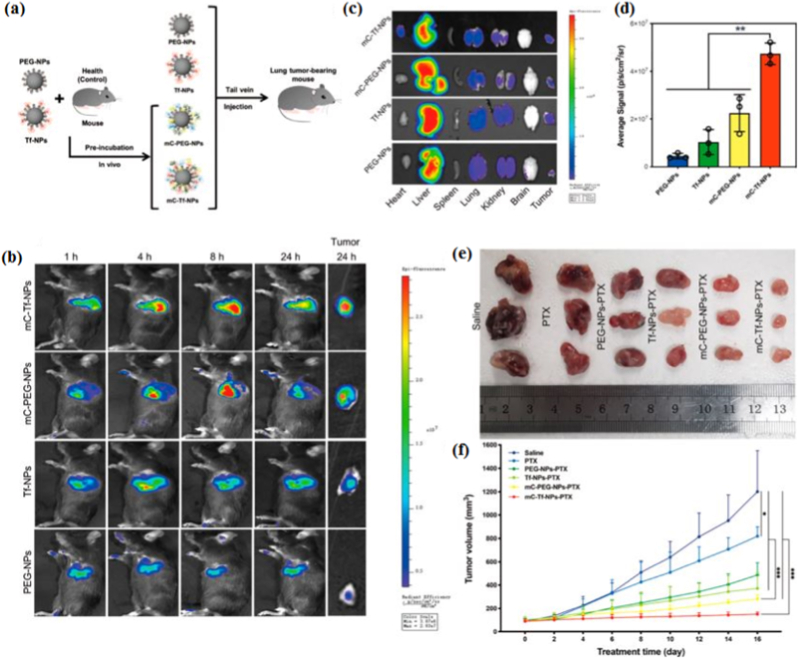
Biodistribution of DiD-loaded PEG-NPs, Tf-NPs, mC-PEG-NPs, and mC-Tf-NPs. (a) Study Design for Biodistribution Analysis: A schematic representation outlining the experimental procedure for evaluating biodistribution following intravenous administration in LL/2 tumor-bearing C57BL/6 mice. (b) Fluorescence Imaging of DiD Distribution**:** In vivo fluorescence images were captured at 1, 4, 8, and 24 h after injection to observe DiD distribution. Additionally, ex vivo fluorescence images of tumor tissues were collected 24 h post-injection (n = 3). (c) Ex Vivo Imaging of Organs and Tumor Tissues: Fluorescence images of major organs and tumors were obtained after in vivo imaging at the 24-h mark (n = 3). The sequence of tissues presented includes the heart, liver, spleen, lungs, kidneys, brain, and tumor. (d) Fluorescence Intensity in Tumors: Quantitative analysis of fluorescence intensity in tumor tissues was conducted 24 h following injection (n = 3). (e) Tumor Appearance After Treatment: Representative images of tumors were taken after 16 days of antitumor therapy. (f) Tumor Growth Monitoring: Tumor size was measured throughout the 16-day antitumor treatment period. Data are shown as mean ± SD, and statistical differences were assessed using Student's t-test. Significant differences are indicated as ∗p < 0.05 and ∗∗∗p < 0.001. Reprinted with permission from Ref. [238], Copyright 2020 American Chemical Society.

The biomedical applications of stimuli-responsive nanocarriers in targeted drug delivery, gene therapy, and theranostics reflect advanced strategies for controlled and site-specific therapeutic outcomes [233]. The nanocarriers are specifically engineered to respond to various stimuli, such as pH, redox, and enzyme levels, for precise drug delivery in diseased tissues with reduced systemic toxicity [2,234]. This feature not only enhances drug bioavailability and reduces systemic side effects, but also helps overcome biological barriers, such as the complex and heterogeneous tumor micro-environment [2]. Xiong and colleagues [235] designed pH-responsive, biodegradable hollow manganese Prussian blue nano-systems (HMPBzymes) that facilitate the phenotypic shift of macrophages from the pro-inflammatory M1 type to the anti-inflammatory M2 type, thereby preventing and reversing the progression of osteoarthritis [235]. The mesoporous structure of these nanosystems increases the surface area, enhances the interaction between the catalyst and reactant, improves catalytic efficiency, and boosts the drug loading capacity [235]. Koide and colleagues [236] developed temperature-responsive nanoparticles using poly(N-isopropylacrylamide) (pNIPAm) to capture and release paclitaxel (PTX), a low-molecular-weight anticancer drug. These NPs shrink above their LCST and swell below it, enabling temperature-controlled drug release [236]. The study showed that NPs with a swelling ratio above 1.90 effectively captured PTX and released it when cooled, while those with a lower ratio released melittin [236]. Sullivan et al. [237] developed enzyme-responsive micellar nanoparticles designed for targeted drug delivery to the heart following acute myocardial infarction (MI). These nanoparticles were created using peptide-polymer amphiphiles (PPAs) carrying an MMP inhibitor (PD166793) through ring-opening metathesis polymerization (ROMP) [237]. When exposed to matrix metalloproteinases (MMPs), the NPs aggregated, confirming their enzyme responsiveness. This study presents a promising NP-based approach for delivering small-molecule drugs to the heart, offering new possibilities for MI treatment [237]. Despite these encouraging results, it has not always been possible to obtain optimal therapeutic efficiency from the systems. For example, although it has been possible to improve the accumulation of nanoparticles at tumor locations by developing various strategies for surface functionalization, it has still not always been possible to obtain optimal anti-tumor efficiency because of the high rate of tumor growth [238]. This is because there is a major limitation in the conventional approach used for designing nanocarriers, in which optimization is carried out empirically by trial and error. Yu et al. [238] investigated the biodistribution and antitumor efficacy of NPs derived from the blood of healthy mice. As shown in Fig. 4a, mC-Tf-NPs and mC-PEG-NPs were administered to tumor-bearing mice via tail vein injection. In vivo fluorescence imaging (Fig. 4b) revealed that DiD-labeled NPs accumulated in the tumor site within 1 h, with signals persisting for 24 h [238]. Notably, the mC-Tf-NPs group displayed stronger fluorescence signals at 2, 4, and 8 h, suggesting that plasma-derived coatings may enhance tumor targeting and extend NP circulation, especially with Tf ligands [238]. Ex vivo imaging (Fig. 4b and c) confirmed greater NP accumulation in tumors for mC-Tf-NPs groups, while the liver showed the highest NP distribution, indicating its role in NP metabolism. Based on these findings, paclitaxel (PTX) was loaded into NPs to evaluate antitumor efficacy. As depicted in Fig. 4e and 4f, although PTX treatment reduced tumor size by 31.8% compared to saline controls, tumor growth remained rapid in both groups [238]. Critically analyzing the above outcomes, the following deficiencies have been noted that can be attributed to classical experimental approaches to optimization of nanoparticle behavior within the body: insufficient tumor delivery of nanoparticles, unintended uptake by other organs (e.g., liver uptake) and, insufficient treatment efficacy. With regard to the above limitations, an AI-based approach can help develop a more systematic strategy for optimizing the behavior of nanoparticles. In particular, a ML algorithm can analyze the characteristics of nanoparticles (size, charge, ligand concentration, and composition) together with biological outcomes (biodistribution patterns and tumor accumulation) and provide information on the optimal set of design characteristics for maximizing tumor delivery and reducing hepatic uptake. Moreover, ML algorithms can facilitate the selection of optimal ligand functionalization (e.g., concentration of transferrin) and drug loading strategies for better treatment outcomes than those obtained via classical experimentation.

**Fig. 5:**
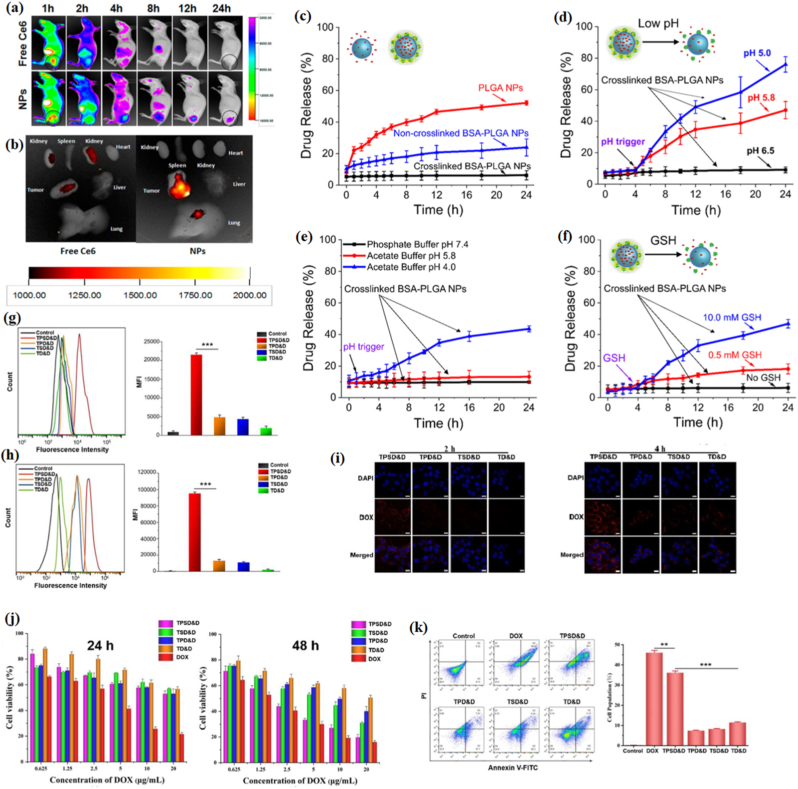
(a) In vivo fluorescence imaging shows the distribution of free Ce6 solution and Dox/Ce6 nanoparticles (NPs), with the black circle highlighting the tumor site. (b) Ex vivo fluorescence imaging of excised tumors and organs from Balb/c nude mice with MCF-7 tumors reveals the localization of NPs 24 h after injection. The drug release profiles of BSA-PLGA NPs were evaluated under different conditions. Fig. 5 (a, b) reprinted with permission from Ref. [239], Copyright 2016 American Chemical Society. (c) In the absence of a stimulus, Dox-TPP release was slower from BSA-PLGA NPs, especially with a crosslinked shell, compared to PLGA NPs. (d, e) Under acidic conditions, both Dox-TPP and rhodamine B showed increased release from crosslinked BSA-PLGA NPs, with greater release at lower pH levels. (f) The presence of glutathione (GSH) also enhanced Dox-TPP release, indicating a reduction-responsive mechanism. Fig. 5 (c, d, e, f)) reprinted with permission from Ref. [240], Copyright 2020 Springer Nature. (g) Fluorescence intensity distribution and histograms show the uptake of micelles by 4T1 cells after 2 h of incubation. (h) After 4 h, the fluorescence intensity increased, indicating greater micelle internalization over time. (i) Confocal laser scanning microscopy (CLSM) images show 4T1 cells incubated with various micelles for 2 and 4 h**,** revealing increased micelle uptake over time. The scale bar represents 25 μm**.** (j) Cytotoxicity studies evaluated the effect of TPSD&D, TSD&D, TPD&D, TD&D micelles, and free DOX on 4T1 cells after 24 and 48 h of incubation, showing the dose- and time-dependent cytotoxic effects of each formulation. (k) Flow cytometric analysis assessed cell apoptosis in 4T1 cells treated with TPSD&D, TSD&D, TPD&D, TD&D micelles, and free DOX using annexin V-FITC/PI staining. The data are presented as the mean ± SD (n = 3), with statistical significance indicated by ∗p < 0.05, ∗∗p < 0.01, ∗∗∗p < 0.001. Fig. 5 (g, h, i, j, k) reprinted with permission from Ref. [241], Copyright 2023 American Chemical Society.

Zhang et al. [239] examined the in vivo distribution of carrier-free nanoparticles and free Ce6 under physiological conditions. While both formulations were widely distributed at early time points, the NPs showed prolonged retention at the tumor site after 12 and 24 h, likely due to improved circulation time and the enhanced permeability and retention effect (Fig. 5a). Ex vivo imaging confirmed higher drug accumulation in tumors for the NPs group (Fig. 5b) [239]. The NPs also displayed different organ distribution, accumulating in the lungs rather than the kidneys like free Ce6. Factors such as pH and body temperature may influence NP stability and drug release, especially in an acidic tumor microenvironment, contributing to their superior delivery efficiency [239]. Palanikumar et al. [240], investigated the drug release behavior of crosslinked BSA-PLGA nanoparticles (NPs) under various conditions. Fig. 5c demonstrates an initial burst release of Dox-TPP from un-crosslinked PLGA NPs within the first hour, attributed to the dissociation of surface-bound drug molecules. This was followed by a sustained release over 24 h due to a combination of drug diffusion and PLGA degradation [240]. In comparison, un-crosslinked BSA-PLGA NPs exhibited a slower release rate, indicating that the BSA shell partially inhibits drug leakage (Fig. 5d). The study [240] also revealed that drug release was pH-dependent, with minimal leakage at neutral or mildly acidic pH (6.5–6.9) but significantly increased release in more acidic conditions (pH 5.0), mimicking the tumor microenvironment (Fig. 5e). This crosslinked structure is essential to prevent premature drug release and ensure effective delivery to target cancer cells [240] (Fig. 5f). From the critical point of view, these studies highlight the following key design principles: the need for structural stability, the role of surface modification, and the importance of stimulus responsiveness in the design of efficient drug delivery systems. These studies also point to the limitations of the conventional design approach in terms of uncontrolled burst release, suboptimal biodistribution, and the difficulty in simultaneously optimizing multiple parameters. Here, the AI-assisted approach offers an exciting solution in terms of the prediction and optimization of the complex relationships between nanoparticle structure, the external conditions, and the release kinetics. The ML approach can be used to predict the release kinetics in terms of the density of cross-linking agents, the nature of the polymers used, and the pH conditions. This approach would minimize the trial and error in experimentation. This would allow the rational design of nanocarriers with improved stability, targeting efficiency, and release kinetics, thus improving the efficacy of the drugs delivered.

In gene therapy, multi-stimuli responsive nanocarriers protect genetic materials (such as Small interfering RNA (siRNA) and DNA) from enzymatic degradation, while ensuring targeted delivery into cells [[242], [243], [244]]. By employing intelligent mechanisms that respond to intracellular stimuli, these systems enable controlled release of genetic payloads at specific sites, which is crucial for treating genetic disorders and cancers [3,245]. Chen et al. [241] evaluated the antitumor activity and cellular uptake of TPSD&D, TPD&D, TSD&D, and TD&D micelles. As shown in (Fig. 5j), TPSD&D micelles exhibited the highest cytotoxicity against 4T1 cells (79.2% lethality after 48 h) due to their dual responsiveness to MMP2/9 enzymes and intracellular GSH. Fig. 5i indicate that TPSD&D micelles had the highest cellular uptake, nearly doubling from 2 to 4 h. Fig. 5g and h further confirm this increased uptake, with TPSD&D micelles showing the strongest fluorescence intensity. In Fig. 5k, these micelles also induced the highest apoptosis rate (36.1%), significantly outperforming other groups, highlighting their superior antitumor efficacy. From a design perspective, this research underscores the vital role of multi-stimuli responsiveness in surmounting biological challenges and improving therapeutic potency. This is underscored by the combination of enzyme sensitivity and redox-mediated release, which enables nanocarriers to remain stable during circulation while becoming highly responsive in the target cellular microenvironment. However, the enhanced complexity of these nanocarriers poses challenges in terms of replicability, scalability, and simultaneous optimization of all relevant parameters. However, through critical evaluation, several issues can be identified when using traditional nanocarriers for targeted drug delivery, which include uncontrolled burst release, off-target accumulation in other organs, and the complexities involved in designing multiple stimuli-responsive systems. In this regard, AI-enabled techniques may present a superior approach towards overcoming such obstacles. Specifically, the application of AI techniques through ML algorithms to analyze the structural properties of nanoparticles (such as cross-linking, polymer type, and surface coating) as well as environmental factors (such as pH and temperature) may enable predictions of the rate of release and identification of designs with minimum levels of burst release. In addition, the integration of physicochemical properties (such as size, surface charge, and hydrophobicity) and biodistribution results from in vivo experiments would allow prediction and minimization of off-target accumulation, organ accumulation, such as lung or kidney uptake observed in these studies. With respect to multi-stimuli responsive vectors for gene delivery, AI could play an even greater role in helping to optimize the combination of stimuli sensitive components (such as cleavage by enzymes and redox responsive components) through predictive models of their interaction. This would be especially useful for cases such as the TPSD&D micelles where several parameters need to be optimized for high cytotoxicity and efficient cell uptake. In general, the use of AI technology will allow moving beyond a trial and error approach towards predictive engineering that will allow us to consider problems like premature drug release, poor distribution, and design complexity ahead of time.

In theranostic applications, these nanocarriers not only facilitate effective drug delivery but also enable simultaneous diagnosis and real-time monitoring of disease progression [246,247]. For example, pH- and redox-responsive nanoparticles can provide controlled drug release, while serving as contrast agents for magnetic resonance imaging (MRI) [247,248] or enhancing photothermal therapies [249]. Such multifunctional systems enhance the ability to personalize the treatment and contribute to the development of innovative approaches in precision medicine, paving the way for more effective and tailored therapeutic interventions [249]. AI has emerged as a useful tool in the rational engineering of nanomaterials for drug delivery, having supplanted the traditional empirical approach in favor of a predictive approach. This is particularly important in the context of the vast design space of nanocarriers, where small changes in size, shape, surface chemistry, or functionalization can lead to large differences in pharmacokinetics and therapeutic efficacy [250]. By leveraging the strengths of ML, DL, and advanced computational modeling, AI platforms enable the systematic exploration of this complex and high-dimensional design space. Such platforms can uncover hidden relationships between structure and properties, point out non-intuitive trends in performance, and shortlist promising formulations with superior therapeutic properties [226]. Therefore, AI-assisted workflows promote the optimization of experiments, lower development expenses, and improve the probability of a successful clinical translation by bridging the gap between preclinical results and practical implementation [251]. AI advances four closely interconnected fields. The first field is design and screening for predicting models that estimate essential physicochemical properties, such as particle size distribution and surface charge, employing ML and ensemble learning strategies. The second field is the use of DL algorithms and multimodal data integration that enable the interpretation of non-linear correlations between synthesis variables (pH, temperature, concentration, and reaction time) and functional properties. The third area is Optimization and discovery to generate frameworks, such as GANs, VAEs, and RL, which allow for the exploration of unexplored chemical and structural space. The final area is Clinical translation and regulation to XAI methods and data governance strategies improve model transparency, reproducibility, and regulatory confidence (Table 5).

**Table 5:** Roles of AI throughout the development phases of nanomaterial-based drug delivery systems.

Resulting Impact	Computational platforms/Data Resources	Methodological Framework	AI- Driven Function	Research Phase
Accelerated candidate prioritization	NanoCommons	ML-based predictive models	Estimation of key physicochemical attributes	Design and Pre-screening
Mechanistic Interpretation-Hypothesis development	caNanoLab	DL architectures/Integration frameworks	Identification, Synthesis-Function relationships	Mechanistic Elucidation
Proposed formulations with improved functional characteristics	Nanomaterial Registry	GAN, VAE, RL	Identification of new chemical entities	Design optimization and discovery
Increased regulatory trust and reduced development time and cost	NanoSolveIT/FDA Predictive Toxicology Roadmap	XAI, data governance	Enhancement of reproducibility and model transparency	Clinical Implementation & Regulatory Integration

In conclusion, Thus, in summary, the above analysis of the recent research works shows that the effectiveness of stimuli-responsive nanocarriers in biomedical applications depends not only on the stimuli-responsiveness to a single stimulus, but also on the rational control of several other parameters [214]. Throughout targeted drug delivery, gene therapy, and theranostic medicine, a common theme in the design of nanocarriers is apparent: multi-functionality and multi-responsiveness are beneficial for nanocarriers, but they have led to great complexity in the optimization of nanocarriers. Although traditional methods of trial and error have led to significant advancements in the field, they have shown themselves to be deficient in efficiently traversing the high-dimensional space of nanocarriers and determining the optimal combination of properties [252]. In this context, AI-based strategies confer a paradigm-shifting advantage through the ability to perform predictive modeling of structure-property-function relationships, revealing non-intuitive design paradigms, and performing multi-objective optimization. Compared to traditional methods, AI-based frameworks offer considerable advantages with respect to reduced experimental burden, reproducibility, and speed to identify high-performance nanocarrier systems. Notably, AI is especially useful in complex nanoscale platforms such as theranostic and gene delivery systems, where several competing factors must be optimized simultaneously [253]. The incorporation of AI in nanocarrier design takes this progress to the next level by offering data-driven optimization, modeling, and exploration of complex design spaces. AI-assisted approaches optimize targeted drug delivery, gene therapy, and theranostic development by accelerating material screening, reproducibility, and design decisions [251]. Despite these advances, challenges persist, including the requirement for high-quality data, better model interpretability, and validation schemes to facilitate the successful translation of in silico-based predictions into experimental and clinical reality. For nanomedicine, it will be crucial to overcome these challenges in order to successfully harness the full potential of AI-based nanomedicine. It is important to note that, as opposed to traditionally empirically designed systems, there is a clear advantage in using AI algorithms to solve these problems because traditional approaches consider optimizing one variable after another, without taking into account potential nonlinear relations between such parameters as chemical composition, responsiveness to a certain kind of stimuli, and biological impact. However, ML models enable the simultaneous analysis of several descriptors at once, which makes it possible to make predictions about drug delivery rate, cellular uptake efficiency, and other relevant aspects, identifying new high-performing but unexpected formulations, thus reducing the volume of experiments and increasing their reproducibility. AI algorithms also make it possible to design more advanced multifunctional systems that respond to more than one external factor at a time.

However, despite the advantages, there are challenges associated with translating research results into clinical applications, namely differences between the in vitro and in vivo performance of such systems, the lack of data for training models, and the necessity of designing models with clear interpretability.

## 5 Challenges and future perspectives

Notwithstanding the swift integration of AI into DDS design, there are technical, clinical, and translational issues that need to be addressed in a systematic manner to facilitate effective real-world implementation. One of the major technical hurdles is the lack of data. There is a lack of high-quality, standardized data that correlates material composition, synthesis conditions, physicochemical characteristics (such as particle size, polydispersity index, and zeta potential), and in vivo performance. Most of the existing data is small, laboratory-specific, and not uniform in experimental conditions [254]. Moreover, the lack of metadata, non-uniform reporting, and batch-to-batch differences make it difficult to integrate studies. Another important limitation is the interpretability and robustness of the model. Although the DL models are able to learn the highly nonlinear synthesis-performance relationships, the black-box nature of these models has raised concerns about their mechanistic understanding and regulatory approval. Although XAI techniques have helped to overcome this problem to some extent, the problem of interpretability-performance trade-offs is still open. Moreover, model overfitting, lack of external validation, and lack of prospective experimental validation often make it difficult to deploy the models effectively. The problem of high dimensionality of the formulation space is also a computational challenge. Nanocarrier formulations are often complex and consist of multiple components. A full search of this design space is computationally intensive, especially when applying reinforcement learning (RL) or generative approaches such as GANs and VAEs. In addition, generative approaches could generate candidates that are chemically impossible or synthetically impractical without the inclusion of domain knowledge. Finally, the integration of multimodal data, such as imaging, omics, pharmacokinetics, and biodistribution, is a field that is not well developed [254]. Future DDS research will need to develop multimodal fusion models that can connect molecular-level information with systems-level biological responses.

However, aside from the computational aspects, the complexity of biological systems still poses a challenge. In vitro systems may not be able to accurately reflect the microenvironment of tumors or diseased tissues, which may cause discrepancies between the predicted and actual performance of the system in vivo. AI models trained on simplified systems may not have high predictive validity in real-world applications. Patient variability further adds to the complexity of translation. Differences in immune response, metabolism, and disease progression require tailored delivery systems. Nevertheless, the integration of patient-specific data into AI-assisted DDS development is still in its nascent stages, owing to a lack of access to longitudinal clinical data [254]. From a regulatory point of view, the transparency, reproducibility, and validation criteria of models are highly important. There is an increasing demand from regulatory bodies for explainability and traceability in algorithmic outputs, particularly in the context of AI-assisted formulation development or manufacturing process control. The development of standardized validation pipelines, benchmarking datasets, and reporting formats will be crucial for regulatory approval and industrial acceptance. Even if AI-optimized formulations show better performance in the laboratory, the manufacturability and scale-up of these formulations still pose a substantial challenge. Process parameters optimized in the laboratory may not be readily transferable to industrial-scale production. The integration of process analytical technology and real-time monitoring information into AI models may provide the opportunity for closed-loop optimization. In addition, economic viability must also be taken into account. The cost of large-scale model training, hardware requirements, and interdisciplinarity of expertise may pose a challenge to accessibility, especially for early-stage research infrastructure.

Future advances are likely to require the establishment of high-quality, open-access databases that combine physicochemical, biological, and clinical data. Federated learning methods could potentially facilitate collaborative model development while maintaining data privacy [254]. Hybrid modeling paradigms that integrate physics-informed ML, mechanistic modeling, and data-driven modeling are anticipated to improve interpretability and predictive accuracy. Integration with digital twins for DDS production and patient-specific simulations could potentially speed up translation. Moreover, incorporation of domain constraints into generative AI models will enhance chemical validity and synthetic accessibility. Integration of AI-assisted design with automated high-throughput synthesis and microfluidic systems could potentially enable closed-loop discovery systems. Finally, successful translation into the clinic will necessitate a multidisciplinary effort involving materials scientists, pharmaceutical engineers, clinicians, computational scientists, and regulatory scientists. By addressing these technical, clinical, and translation challenges, AI-assisted drug delivery design has the potential to transition from proof-of-concept to scalable, regulatory-compliant, and patient-specific therapeutic platforms.

## 6 Conclusion

There exist stimuli-responsive nanostructures that can serve as an innovative approach to providing spatial and temporal control of drug delivery due to their response to stimuli from both internal sources (pH, redox potential, enzymes) and external sources (temperature, light, magnetic fields). The use of multiple responsive properties can increase the complexity and effectiveness of such nanostructures within a biological milieu. AI is revolutionizing the design of nanocarriers in that scientists are no longer using trial-and-error strategies but rather rely on data-driven optimization of nanocarrier structures. AI algorithms help map out the relationship between nanostructure characteristics and their function as well as predict optimal carrier design and kinetics of drug delivery based on physiological parameters. However, there are several critical breakthroughs that must be achieved before the practical implementation of AI-guided stimuli-responsive nanocarriers can become a reality. To begin with, the creation of quality databases and rigorous external validation procedures will be fundamental to guaranteeing the accuracy and reliability of the models used. Moreover, the implementation of closed-loop systems that combine AI-powered design with high-throughput experimentation may provide means for the automated optimization of the formulation process. Finally, the inclusion of multimodal patient data and explainable AI methods will be vital to facilitating personalized and adaptive drug delivery protocols.

## Informed consent statement

Not applicable.

## Institutional review board statement

Not applicable.

## Funding statement

The author(s) received no financial support for the research, authorship, and/or publication of this article.

## CRediT authorship contribution statement

**Sara Yazdani:** Conceptualization, Visualization, Writing – original draft, Writing – review & editing. **Mehrdad Mozaffarian:** Project administration, Supervision, Writing – review & editing. **Gholamreza Pazuki:** Conceptualization, Project administration, Supervision, Writing – review & editing. **Naghmeh Hadidi:** Visualization.

## Declaration of competing interest

The authors declare no conflict of interest.

## Data Availability

No data was used for the research described in the article.
